# Multiplexed Imaging Mass Cytometry Reveals Tumor-immune Microenvironment–dependent Hormone Receptor Expression in Adult-Type Ovarian Granulosa Cell Tumors

**DOI:** 10.1158/2767-9764.CRC-25-0333

**Published:** 2025-10-27

**Authors:** Eleonora Y. Khlebus, Veena K. Vuttaradhi, Sammy Ferri-Borgogno, Allison L. Brodsky, Barrett C. Lawson, Samuel C. Mok, R. Tyler Hillman

**Affiliations:** 1 Department of Gynecologic Oncology and Reproductive Medicine, The University of Texas MD Anderson Cancer Center, Houston, Texas.; 2 Department of Anatomic Pathology, The University of Texas MD Anderson Cancer Center, Houston, Texas.; 3 Division of Gynecologic Oncology, Department of Obstetrics, Gynecology and Reproductive Sciences, University of California San Diego, Rebecca and John Moores Cancer Center, La Jolla, California.

## Abstract

Adult-type granulosa cell tumors (AGCT) are rare ovarian tumors with few effective treatments for recurrent disease. To elucidate spatial features and cellular interactions within the AGCT tumor microenvironment, we applied imaging mass cytometry using a 34-marker panel on 130 regions from 24 AGCT samples, profiling more than 900,000 single cells. Analysis confirmed the immune “cold” phenotype of AGCTs and showed higher macrophage abundance in recurrent compared with primary tumors. We observed substantial heterogeneity in tissue architecture across samples, including variable presence of FOXL2+ cells embedded in collagen-rich regions (FOXL2+COL1A1+ cells). Based on tumor microenvironment composition, we defined two AGCT subtypes: AGCT-1 and AGCT-2 with distinct FOXL2+ cell distributions, differences in progesterone receptor expression, and unique transcriptomic profiles. Our findings highlight the role of macrophages, Foxl2+ subpopulations, and the extracellular matrix in AGCT progression and suggest AGCT subtype–specific vulnerabilities that could inform personalized therapies for this rare malignancy.

**Significance:**

We discovered two histologically and molecularly distinct forms of AGCTs that differ in cell composition, immune activity, and hormone signals. These findings point to new opportunities for more personalized treatment of this rare ovarian cancer.

## Introduction

Adult-type granulosa cell tumors (AGCT) are rare ovarian sex cord–stromal tumors, accounting for 2% to 5% of all ovarian cancers, with an annual incidence of approximately 0.5 to 1.0 per 100,000 women (1, 2). Recurrent AGCTs are nearly always incurable due to limited effective systemic therapies, and recurrence is difficult to predict, making its prevention and management a significant clinical challenge. Treatment of recurrent AGCT often involves surgical cytoreduction procedures (3), combined with systemic chemotherapy (4–7) or hormone-based therapy (8, 9). However, these approaches generally yield low objective response rates, and clinical molecular profiling has not identified a high frequency of actionable genetic alterations in AGCT (10–13). Due to the rarity of AGCTs, studies and clinical trials specifically designed for the treatment of AGCT are limited, resulting in a lack of effective, evidence-based treatment options.

The tumor microenvironment (TME) plays a critical role in cancer progression and treatment response in different cancer types (14). However, in AGCTs, comprehensive characterization of the TME, especially its spatial organization, remains limited because of low tissue availability. In our previous work, we characterized the AGCT TME using bulk RNA sequencing (RNA-seq) data of cryopreserved tissue samples and computational TME deconvolution methods (15), which provided a general overview of TME cell subtypes but lacked precise cell identification and spatial resolution. A spatially resolved analysis of the AGCT TME has not yet been reported.

To address this unmet need, we applied imaging mass cytometry (IMC; ref. 16) to formalin-fixed, paraffin-embedded (FFPE) AGCT tissue samples. IMC allows high-dimensional, multiplexed, single-cell analysis with spatial resolution using isotope-conjugated antibodies and has proven effective in profiling primary and relapsed tumors in hepatocellular carcinoma (17), brain tumors (18), and Hodgkin lymphoma (19). Using a customized 34-marker IMC panel, we examined 24 AGCT samples (8 primary and 16 recurrent, nonmatched tumors) that have been extensively characterized (10, 15) and profiled more than 900,000 individual cells and their spatial context. This analysis revealed the presence of FOXL2+ cells embedded in collagen-rich areas, which we called FOXL2+COL1A1+ cells. Presence of those cells in AGCT tissue samples correlated with distinct stromal composition and cell interactions patterns, suggesting a pivotal role in AGCT pathology.

IMC-based investigation of the AGCT TME revealed a high degree of tissue structure heterogeneity between AGCT samples, yet also uncovered cross-cutting similarities between primary and recurrent tumors. In other more common cancers such as breast (20), ovarian (21), and non–small cell lung cancers (22), histologic and molecular subtypes are established and clinically informative. Such classification has been lacking in AGCT due to its rarity. Using IMC-based approach, in this study we proposed to identify two distinct TME subtypes: AGCT-1 and AGCT-2, characterized by different fractions of FOXL2+COL1A1+ cells. These subtypes exhibited greater transcriptomic differences than the division into primary versus recurrent tumors, supporting the idea that intrinsic TME features, which can be seen on histology with IMC, more accurately reflect underlying molecular differences. Importantly, we also observed distinct expression patterns of hormone receptors, including progesterone receptor (PR) and estrogen receptor α (ERα), with PR+ and ERα+ cell populations varying between AGCT-1 and AGCT-2. These differences may have implications for hormone-targeted therapy and resistance to commonly used endocrine treatments in AGCT.

In summary, we present the first spatially resolved, deep TME characterization of a relatively big AGCT cohort by IMC. We propose a pivotal role for FOXL2+COL1A1+ cells in AGCT pathology and define two histologically and molecularly distinct AGCT subtypes with potential clinical relevance. Our findings highlight the importance of incorporating TME-based subtyping into treatment planning and open new avenues for biomarker development and targeted therapies in this rare ovarian cancer.

## Materials and Methods

### Patients and samples

FFPE tissue samples from a total of 24 patients with pathologically confirmed AGCT were obtained from The University of Texas MD Anderson Cancer Center Multidisciplinary Gynecologic Cancer Tumor Bank. This cohort included 8 primary and 16 nonmatched recurrent tumors. All patients provided written, informed consent for biospecimen acquisition under an Institutional Review Board–approved protocol (LAB02-188). The specific use of these samples for the research described in this article was separately Institutional Review Board–approved (protocols PA16-0891 and 2022-0547). Previously, these AGCT samples were described and analyzed by whole-exome sequencing (10) and total RNA-seq (15). The somatic FOXL2 c.C402G mutation was identified in 23 of 24 tumor samples as mentioned before (10). Samples from primary AGCTs were all derived from ovarian sites, whereas recurrent tumors originated from the abdomen pelvis, large bowel, liver, ovary, and spleen. The clinical summary for the studied cohort was presented in our previous works (10, 15), and detailed clinical characteristics for each patient are available through the Zenodo repository linked to this study (https://doi.org/10.5281/zenodo.15384724).

### Tissue processing and IMC staining

The AGCT tissue sections were prepared at the departmental pathology core at MD Anderson Cancer Center. Each tissue was subjected to a series of seven serial sections of 5 μm thickness, labeled from slide #0 to #6, and stored inside a desiccator slide chamber at −80°C until stained. Slides #1 and #2 were used for hematoxylin and eosin (H&E) and IMC staining, respectively, whereas all other sections were stored as backups. Conventional H&E-stained slides were used by an expert pathologist (B.C. Lawson) to annotate tumor regions and validate overall tissue morphology prior to IMC analysis.

Before staining with metal-conjugated antibodies, the tissue sections were deparaffinized by placing them in an oven at 60°C for 1 hour, followed by heat-inducible antigen retrieval using EZ-AR1 Elegance Citra pH 6 buffer (cat. # HK546-XAK, BioGenex) in the BioGenex Microwave (EZ-RT system, MW016-IR or MW015-IR) for one cycle at 107°C for 15 minutes. Blocking was performed using 100 to 200 μL of UltraVision Protein Block (TA125PBQ, Fisher Scientific), incubated at room temperature for 1 hour. A diluted antibody mix (antibody diluent, S0809 Dako) was added to the slides and incubated at 4°C overnight in a moist chamber to reduce evaporation. The next day, the slides were washed with phosphate-buffered saline with Tween-20 three times for 5 minutes while stirring at a low speed on a rocker, followed by nuclei staining with Ir-intercalator stain for 5 minutes at room temperature. This was followed by additional washes: once with Dulbecco's phosphate-buffered saline with Tween-20 and twice with Dulbecco's phosphate-buffered saline at room temperature on a slow rocker. The slides were then dipped immediately into water, dried in a laminar airflow chamber, and stored at 4°C until image acquisition.

For IMC staining we designed a custom 34-marker panel (32 protein markers and 2 DNA markers). Prior to the final IMC experiments, each marker underwent individual validation to ensure specificity and signal quality. To confirm the immunodetection pattern, antibodies were tested by IHC, and their expression was evaluated by a pathologist. After metal conjugation, antibodies were titrated to get the optimal signal-to-noise ratio. The complete list of antibodies, clones, conjugated metals, and dilutions is included in Supplementary Table S1. After IMC staining, tissue sections were subjected to laser ablation and image acquisition.

### IMC and image acquisition

IMC was performed at the Flow Cytometry and Cellular Imaging Facility at The University of Texas MD Anderson Cancer Center with the Standard BioTools Hyperion XTi Imaging System. For each sample, we selected 1-mm^2^ representative regions of interest (ROI) which contained regions that were representative of the whole range of AGCT pathology, including tumor and tumor–stroma areas. H&E slides were used to guide the selection of ROIs by an expert gynecologic oncology pathologist (B.C. Lawson). Selected ROIs were laser-ablated spot-by-spot at 200 Hz resulting in a pixel size/resolution of 1 mm^2^. Metal-conjugated antibodies were detected using a Hyperion Imaging Mass Cytometer (Standard BioTools). Visualization of images was conducted using MCD Viewer v1.0.560.6 (Standard BioTools).

### IMC data analysis

Files generated in the process of data acquisition (Mcd format) were converted into .tiff and .txt files using MCD Viewer v1.0.560.6 (Standard BioTools) and imported to the Visiopharm software (v2024.07.1.16912). Visiopharm software was used for tissue detection, tissue segmentation into tumor and stromal/collagen-rich areas, cell segmentation, and cell phenotyping. Cell type annotations were subsequently reviewed by a pathologist based on known marker combinations, spatial distribution, and morphologic features. This integrative approach ensured accurate classification of cell populations. To perform cell morphology analysis, we used next morphologic parameters calculated by Visiopharm software based on features of identified cells: convexity, eccentricity, ellipticalness, form factor, largest diameter, lesser diameter, major axis, minor axis, perimeter, and solidity. Further cell coordinates, phenotypes, association with tumor or stromal/collagen-rich area, and morphologic parameters of cells were exported from Visiopharm. All further image analyses and statistical analysis were performed using R software (v4.4.2). A *P* < 0.05 was considered significant if not mentioned otherwise.

Dimensionality reduction and visualization of high-dimensional IMC data were performed using t-distributed stochastic neighbor embedding (t-SNE; ref. 23) implemented in the Rtsne R package (v0.17). The following parameters were used for all t-SNE analyses: perplexity = 30, θ = 0.5, and dims = 2. For the global t-SNE map of major cell types, six major cell groups were considered. From each group, 1,000 cells were randomly selected, resulting in a balanced dataset of 6,000 cells used for dimensionality reduction. For the focused t-SNE analysis of FOXL2+ cells, eight distinct subpopulations were defined based on PR and ERα expression within FOXL2+COL1A1− and FOXL2+COL1A1+ populations. From each FOXL2^+^ subpopulation, 1,000 cells were randomly selected, totaling 8,000 cells for analysis. Only phenotypic markers were used for the analysis, whereas functional and DNA-associated markers were excluded to emphasize cell identity characteristics.

To perform spatial analysis and calculate cell-to-cell proximity score, we applied a permutation test method implemented as testInteractions function in the R package imcRtools (v1.12.0; ref. 24) as proposed here (25). Using this approach, we determined whether interactions/avoidances between each cell type within each ROI occurred more or less frequently than random observations. Briefly, cells were considered neighbors if the distance between cell centroids was less than 20 μm when the “expansion” interaction graph building method from buildSpatialGraph function in the imcRtools package was used. We also considered 10 nearest neighbors applying the “K-nearest neighbors” method for interaction graph building. For each ROI, a permutation test (*n* = 1,000) of the cell labels was performed to assign a *P* value to each couple of neighboring cells, and here the testInteractions function with method “classic” was applied. Interactions between cells with *P* value < 0.01 were assigned an interaction score (sigval) +1 or −1 according to their enrichment or depletion compared with randomized interactions, whereas a score of 0 was assigned to nonsignificant interactions. The resulting interaction scores were averaged over the all ROIs from each sample following averaging by primary and recurrent AGCT conditions and then represented in heatmaps using ggplot2 (v3.5.1) R package.

### RNA-seq data processing and analysis

For RNA-seq and IMC, adjacent tissue sections from the same tumor blocks were used to ensure consistency. RNA-seq and data processing were performed as described previously (15). Aligned reads for RNA-seq data used in this study are available through the European Genome-Phenome Archive (EGA) under accession EGAS00001006478. Differential gene expression analysis was performed using R package “DESeq2” [v.1.45.3; (26)]. Multiple comparisons adjustment was performed using the Benjamini–Hochberg approach, and differentially expressed genes (DEG) with the adjusted *P* value of <0.05 and absolute fold change (FC) >2 were considered statistically significant. Functional enrichment analysis were performed using the “clusterProfiler” R package [v.4.0.5; (27)] with enrichGO function for overrepresentation analysis of gene ontology (GO) terms and gene set enrichment analysis (GSEA) function for performing GSEA (ref. 28) of Hallmark gene set from the Molecular Signatures Database (29). GSEA function was run with the following parameters: eps = 0, minGSSize = 10, maxGSSize = 500, and pvalueCutoff = 0.05; genes were ranked by FC calculated by DESeq2 (AGCT-2 vs. AGCT-1); 1,000 permutations were performed to obtain the randomized enrichment score and calculate the normalized enrichment score; and the *P* value associated with each gene set was adjusted by the Benjamini–Hochberg multiple testing procedures.

### Statistical analysis

Comparisons between continuous AGCT and AGCT subtypes were performed using the Wilcoxon rank-sum test. For each sample, the mean value across all available ROIs was calculated prior to comparison. These sample-level means were then used in the statistical analysis. All tests were two-sided, and *P* values less than 0.05 were considered statistically significant. Statistical analyses were conducted using R v4.4.2.

## Results

### Development of the AGCT IMC panel

**Figure 1.**
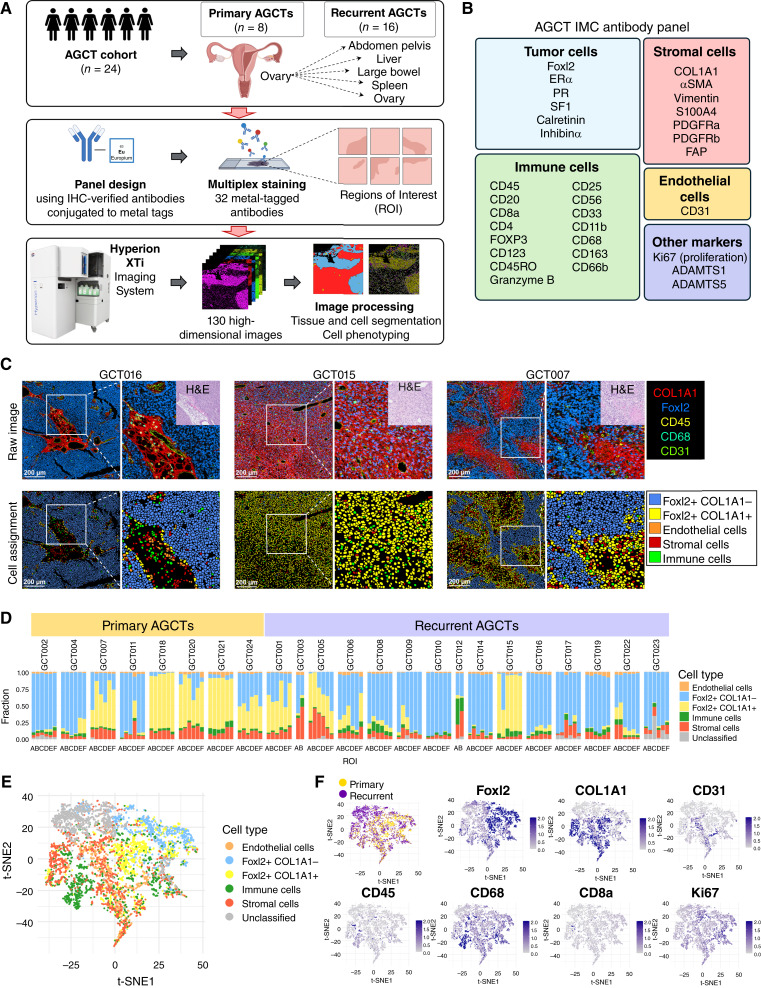
Overview of the clinical cohort, project workflow, panel design, and major cell types presented in the AGCT TME. **A,** Overview of the clinical cohort and experimental workflow. The study includes primary (*n* = 8) and nonmatched recurrent (*n* = 16) AGCT tissue samples (recurrences are collected from five different sites: abdomen pelvis, spleen, liver, large bowel, and ovary). Tumor tissue was removed during surgery, followed by fixation, paraffin embedding, and sectioning. The 32-antibody panel was designed and used for staining 1-mm^2^ ROIs of AGCT FFPE tissue samples targeting tumor, stromal, and immune cell phenotypes. In total 130 high-dimensional images were acquired by IMC. The resulting images were processed and analyzed using bioinformatics tools. Cell segmentation allowed the analysis of marker expression in each individual cell, providing the data for single-cell analysis. **B,** Markers used for the AGCT IMC antibody panel and targeted cell types. **C,** Representative IMC images from three AGCT samples (top) and corresponding cell assignment (bottom), with magnified regions to the right of each image. The color codes for IMC markers (top right) and cell assignment (bottom right) are provided. Scale bars, 200 μm. **D,** The distribution of major cell groups as a percentage of all cells in the TME, highlighting the high presence of FOXL2+ cells and the “cold” immune phenotype of AGCT samples. Each vertical bar represents an individual image (*n* = 130 images), called the ROI sorted by sample, grouped by primary (left) and recurrent (right) AGCT conditions. Colors correspond to major cell types identified in the AGCT TME. **E,** t-SNE dimensionality reduction map representing the major cell types identified in the AGCT TME, with each color corresponding to a cell type and each dot representing a single cell. **F,** t-SNE map overlaid with different AGCT conditions (yellow, primary tumors; purple, recurrent tumors) and with the expression intensities of FOXL2, COL1A1, CD31, CD45, CD68, CD8a, and Ki67.

The purpose of this investigation was to identify spatial features of the AGCT TME using a well-characterized cohort of 24 patients with AGCT: 8 primary and 16 nonmatched recurrent tumors (Fig. 1A). We have previously analyzed the TME of this cohort using bulk RNA-seq data and computational deconvolution methods (15), but this approach lacks spatial resolution and has reduced sensitivity due to reliance on bulk averaging of gene expression signal. Moreover, gene expression may not accurately reflect the actual presence of protein products in the TME, which may be diffusible or otherwise secreted into extracellular spaces. In this study, we used highly multiplexed IMC to comprehensively profile not only the cellular composition but also the spatial organization of the AGCT TME. To do this, we designed and validated a custom antibody panel specific to AGCT biology to detect 34 different markers (32 protein markers and 2 DNA markers). The 34 markers in our custom panel were selected to comprehensively profile the cellular and molecular landscape of AGCT tumors and their microenvironment based on both prior knowledge and our previous computational studies (15).

Our panel included markers of tumor cells [FOXL2, ERα, PR, steroidogenic factor 1 (SF1), calretinin, and inhibin α (inhibinα)]. Specifically, FOXL2 was included as a key marker for AGCT tumor cell detection, with SF1, calretinin, and inhibinα serving as additional controls to validate AGCT cell staining. ERα and PR were incorporated given their importance in hormonal therapy and potential therapeutic response. To better understand the TME, we included multiple stromal markers [COL1A1, α-smooth muscle actin (αSMA), vimentin, S100A4, PDGFRa, PDGFRb, and fibroblast activation protein (FAP)] to identify and differentiate stromal cell subtypes. This selection was motivated by our previous computational findings showing differences in stromal cell abundance between primary and recurrent AGCTs (15); in this study, we aimed to pinpoint specific stromal subpopulations that may differ and could represent therapeutic targets. Additionally, several immune cell markers were added to profile the key immune cell subsets present in AGCT samples (CD45, CD20, CD8a, CD4, FOXP3, CD25, CD56, CD33, CD11b, CD68, CD163, CD66b, CD123, CD45RO, and granzyme B). Other included markers were ADAMTS1, ADAMTS5, the proliferation marker Ki67, and the endothelial cell marker CD31 (Fig. 1B). The two DNA markers served as double controls for nuclei identification and played important technical roles: they enabled reliable detection of cell nuclei for accurate cell segmentation and allowed to assess cellular morphology and tissue architecture within the imaging data. A table with marker description is provided as Supplementary Table S1.

After inspection, all 34 markers from this panel demonstrated staining consistent with expected patterns of morphology and subcellular localization (Supplementary Fig. S1A). As expected for AGCT, our IMC data showed strong nuclear FOXL2 expression across all tumor cells (Supplementary Fig. S1B). SF1 was also expressed in most AGCT tumor samples, though its staining seemed more diffuse than FOXL2; therefore, FOXL2 was used as the primary marker for tumor cell identification. We observed good overlap between nuclear FOXL2 and ER/PR staining, supporting the reliability of the ER and PR signals detected by IMC. Although only six AGCT samples showed more than 5% of FOXL2+ cells co-expressing ER and we observed diffuse expression in some cases, the staining seemed accurate. Most AGCT samples also expressed CD56, and as many CD56 isoforms are membrane proteins, the staining appeared broadly and evenly distributed across tumor tissue, as expected (Supplementary Fig. S1B).

For each AGCT FFPE tissue section, we selected up to six 1 × 1 mm ROIs per tumor, as chosen based on H&E review by an expert gynecologic oncology pathologist (B.C. Lawson). Two recurrent tumor samples were too small to acquire six nonoverlapping ROIs, so two ROIs were used instead. One recurrent tumor tissue sample was lost during tissue processing. In total, we acquired 130 high-dimensional histopathology images representing 8 primary (48 images) and 15 nonmatched recurrent tumors (82 images). We segmented a total of 943,344 single cells across all 130 images (mean = 7,256 cells per image). For cell phenotyping, we then used a supervised lineage assignment approach based on marker expression to classify tumor cells, stromal cells, blood vessels, and immune cell populations using canonical immune cell markers (Fig. 1B and C; Supplementary Fig. S2). Overall, we assigned 98% of segmented cells (*n* = 928,567 single cells) to one of distinguishable cell classes.

As expected, FOXL2+ cells represented a major cell population across all AGCT tissue samples (Fig. 1C and D), confirming the initial diagnosis that all studied cases are indeed AGCT. Of note, in some samples, we observed numerous FOXL2+ cells deeply embedded within collagen-positive regions. We refer to these as FOXL2+COL1A1+ cells (highlighted in yellow in Fig. 1C–E), and they are examined in greater detail later in this study. The next most prevalent cell populations were stromal cells, whereas immune cells and endothelial cells were relatively infrequent, highlighting the “cold” immune phenotype of AGCT samples and the low levels of vasculature overall (Fig. 1D). Despite high architectural heterogeneity between AGCT samples, we observed that the cell composition was generally consistent across ROIs from the same tumor sample (Fig. 1D). In general, major cell types were equally distributed in primary and recurrent tumors, although we observed a trend toward less abundance of FOXL2+COL1A1+ cells in recurrent tumors (Supplementary Fig. S3; *P* = 0.056).

In order to examine the common distribution characteristics of the AGCT TME, we randomly selected 1,000 cells from each major cell group presented on Fig. 1D and conducted a t-SNE analysis based on expression of multiple phenotypic markers (Fig. 1E and F; Supplementary Fig. S4). In this space, we identified distinguishable cellular clusters with distinct expression profiles. A t-SNE map overlaid with AGCT primary and recurrent conditions showed matching, which suggested minimal difference in major cell types’ composition between primary and recurrent AGCTs (Fig. 1F). A t-SNE map of FOXL2+COL1A1− and FOXL2+COL1A1+ cells showed these to be separated as distinct cellular clusters (Fig. 1E, blue and yellow colors, respectively). Expression of CD68 was identified in most immune cells (Fig. 1E and F), suggesting that macrophages represent the most abundant immune cell type in AGCTs.

### AGCT contains distinct FOXL2+ subpopulations defined by local collagen composition

**Figure 2.**
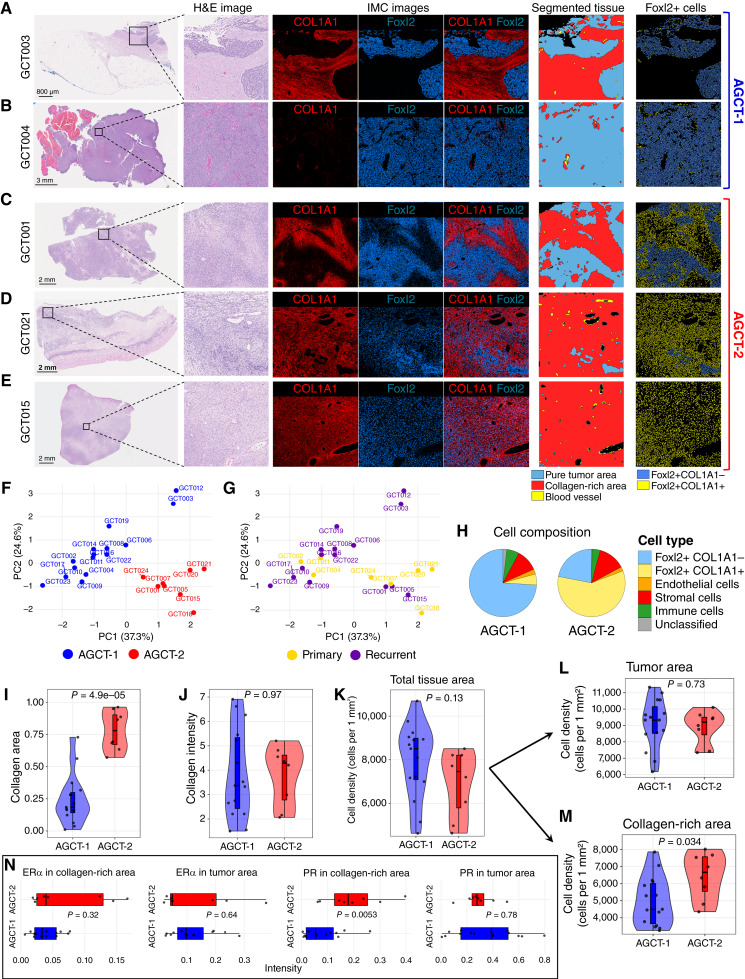
The AGCT TME exhibits two distinct patterns of tumor/stromal structure. **A–E,** Representative IMC images, adjacent H&E images, segmented tissue masks, and segmented FOXL2+ cell masks. **A,** Sample with highly differentiated tumor and stroma, a clear distinguishable border between COL1A1+ and FOXL2+ staining areas and small fraction of FOXL2+COL1A1+ cells. **B,** Samples mostly contained pure tumor areas with low stromal content. **C,** Sample with poorly demarcated tumor–stroma boundaries and high fraction of FOXL2+ cells in a collagen-rich area. **D,** Sample with a large stromal area and relatively small highly distinguishable pure tumor islands. **E,** Samples with significant fibromatous morphology. **F,** PCA plot representing AGCT samples’ relation based on the composition of major cell types identified in the AGCT TME. The colors indicate 15 AGCT samples representing AGCT-1 subtype with the FOXL2+COL1A1+ cell fractions (blue) and eight AGCT samples representing AGCT-2 subtype with high FOXL2+COL1A1+ cell fractions (red). **G,** PCA plot overlaid with AGCT conditions (yellow, primary tumors; purple, recurrent tumors). The matching suggested minimal difference in major cell types’ composition between primary and recurrent AGCTs. **H,** Cell composition in two AGCT subtypes. **I–N,** Comparison of AGCT-1 and AGCT-2 subtypes: stromal/collagen-rich area fraction in tissue area (**I**); collagen intensity (**J**); cell densities in total tissue area (**K**), tumor area (**L**), and collagen-rich area (**M**); ERα and PR intensities through tumor or collagen-rich areas (**N**). In each ROI, stromal/collagen-rich area normalized per total tissue area in the ROI (**I**). Mean values per sample plotted and used for calculation of statistics. A Wilcoxon test was used to compare differences across two subtypes of AGCT samples. *P* values are displayed on the plots.

Visual inspection of H&E and IMC images revealed a high level of morphologic heterogeneity in AGCT tissue samples in the tumor–stroma ratio, as well as boundaries between tumor and stromal areas. Most samples had more than 50% of pure tumor area, which was FOXL2+COL1A1− and exhibited a distinguishable border between COL1A1+ and FOXL2+ staining areas (Fig. 2A). Some AGCT samples contained only tumor areas with low stromal content (Fig. 2B). Several samples had poorly demarcated tumor–stroma boundaries (Fig. 2C), and others had a large stromal area with relatively small, highly distinguishable pure tumor islands (Fig. 2D). Lastly, three samples displayed significant fibromatous morphology through the whole tumor tissue sample slide (Fig. 2E).

To gain a deeper insight into the TME organization of AGCT, we next segmented the tumor tissue samples into compartments and analyzed the cellular composition within each area separately. Initially, we aimed to define tumor and stromal regions based on FOXL2 and COL1A1 staining, respectively. As expected for AGCT, our IMC data showed robust nuclear FOXL2 staining. However, in addition to FOXL2+ cells with the classic “coffee bean–shaped” AGCT cellular morphology, we also identified a subset of FOXL2+ cells with irregular or spindle-shaped morphology, located within COL1A1+ regions. These cells seemed more elliptical and resembled stromal elements such as cancer-associated fibroblasts (CAF) rather than classic AGCT cells (Supplementary Fig. S5). These FOXL2+ cells in the COL1A1+ regions frequently co-stained with other tumor markers from our IMC panel, including SF1, calretinin, and inhibinα. Their presence in collagen-rich regions raised a critical challenge: how to reliably distinguish tumor from stroma when FOXL2 and other tumor-associated markers are also detected in COL1A1+ areas, or how to differentiate COL1A1+ fibromatous tumor tissue from stromal regions containing FOXL2+ stromal elements (both classified as FOXL2+COL1A1+).

To address this, we applied the next segmentation approach. First, COL1A1 staining was used to identify collagen-rich regions, followed by FOXL2 staining to define pure tumor areas. The final classification consisted of (i) tumor areas (FOXL2+/COL1A1−, considered pure tumor) and (ii) collagen-rich areas, including FOXL2−/COL1A1+ stroma, FOXL2+/COL1A1+ stroma, and FOXL2+/COL1A1+ fibromatous tumor. The small areas that remained outside these segmented regions exhibited positivity for CD31 and were defined as blood vessels. Resulting areas composition through representative ROIs are shown in Fig. 2A–E (segmented tissue column). Hemorrhagic areas were excluded from further analysis, both during ROI selection and tissue detection, based on the absence of nuclear staining, indicating a lack of viable cells.

The initial observation of Foxl2+ cells within COL1A1+ regions led us to further investigate the features that differentiate FOXL2+COL1A1− and FOXL2+COL1A1+ regions in AGCT samples. During cell phenotyping, we highlighted Foxl2+COL1A1+ cells. In some samples, these cells were relatively rare (Fig. 2A and B, right, yellow), whereas in others, they were abundant, accounting for more than 30% of all FOXL2+ cells (Fig. 2C–E, right, yellow). This pattern was observed in both primary and recurrent AGCTs, as well as in tumors collected from ovarian and nonovarian anatomic locations. Hereafter, we defined two AGCT TME subtypes: AGCT-1 and AGCT-2 (Fig. 2A–E).

AGCT-1 (15/23 tumors; 65%) was enriched for tumors with tightly packed “coffee bean–shaped” FOXL2+COL1A1− cells, infrequent FOXL2+COL1A1+ cells, and low collagen invasion in solid tumor area. In contrast, samples in AGCT-2 (8/23 tumors; 35%) contained more FOXL2+ cells with an irregular shape deeply embedded in a collagen-rich area. To classify AGCT samples into subtypes, we used the proportion of FOXL2+COL1A1+ cells relative to the total number of FOXL2+ cells. A threshold of 37% was applied. The threshold was determined based on the bimodal distribution of cell proportions across samples and defined using the valley method, which identifies the local minimum between the two peaks of the density curve (Supplementary Fig. S6). Principal component analysis (PCA) performed using major cell types as input data showed that our sample division into AGCT-1 and AGCT-2 TME subtypes showed superior subgroup discrimination (Fig. 2F) compared with primary versus recurrent AGCT (Fig. 2G) and anatomic site (Supplementary Fig. S7). Quantification of cellular composition in AGCT-1 and AGCT-2 (Fig. 2H) indicated that the difference between defined AGCT subtypes is mostly in FOXL2+COL1A1+ fractions, whereas fractions of other major cell types between two subtypes were similar. Of note, primary AGCTs had a higher percentage of samples defined as AGCT-2 with high presence of FOXL2+COL1A1+ cells (62.5%, 5/8 primary tumors) in comparison with recurrent AGCTs (20%, 3/15 recurrent tumors). When comparing primary versus recurrent tumors, we did not observe a statistically significant difference in either the normalized COL1A1+ area per tissue area in primary versus recurrent tumors (*P* = 0.29) or the intensity of COL1A1 staining (*P* = 0.36, Wilcoxon test; Supplementary Fig. S8). In contrast, when comparing AGCT subtypes, we observed a difference in collagen-rich area between AGCT-1 and AGCT-2, with higher collagen proportion in AGCT-2 (*P* = 4.9 × 10^−5^; Fig. 2I). Collagen intensity did not differ between AGCT-1 and AGCT-2 (*P* = 0.97; Fig. 2J). Interestingly, overall cell density and tumor compartment cell density did not differ between AGCT-1 and AGCT-2 (*P* = 0.13 and *P* = 0.73; Fig. 2K and L, respectively). In contrast, cell density within the collagen-rich areas showed a significant difference between the two subtypes (*P* = 0.034; Fig. 2M). This finding highlights that defined AGCT subtypes differ not only in the presence of FOXL2+COL1A1+ cells but also in spatial composition and organization of the collagen-rich regions, potentially reflecting differences in the structural properties of collagen and the ECM between the subtypes.

Hormonal therapies that modulate estrogen or progesterone signaling are frequently used in the management of relapsed AGCTs (8). To examine potential functional implications of AGCT-1 and AGCT-2 TME-based classifications, we examined overlap with ERα and PR expression in our IMC data (Fig. 2N). ERα expression did not exhibit a statistically significant difference between AGCT-1 and AGCT-2 subtypes, similar to the lack of difference we observed in ERα/PR expression when comparing primary and recurrent AGCTs (Supplementary Fig. S9). In contrast, we found that expression of PR in collagen-rich areas of AGCT-2 was significantly higher than in AGCT-1 (Fig. 2N). The presence of higher PR expression in the FOXL2+COL1A1+ cells characteristic of the AGCT-2 subtype suggests that TME structure may impart distinct properties with respect to hormonal therapy responsiveness.

### Hormone receptor expression differs across subpopulations of FOXL2+ tumor cells

To further investigate associations between TME structure and hormone receptor expression in AGCT, we next performed a detailed analysis of ERα and PR expression across FOXL2+ AGCT tumor cells. For additional validation of PR staining, we compared our IMC results with IHC staining and observed a correlation between results of both methods (Supplementary Fig. S10).

**Figure 3.**
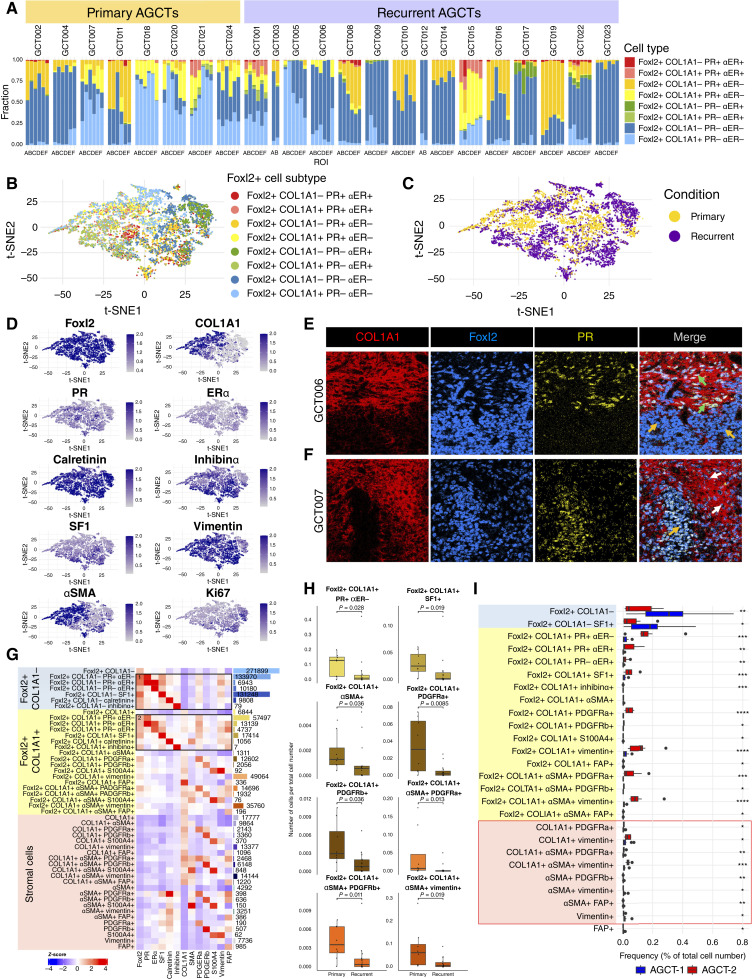
Comprehensive immunophenotyping of FOXL2+COL1A1− and FOXL2+COL1A1+ AGCT regions. **A,** Composition of tumor tissue samples in terms of FOXL2+ cells subpopulations subdivided by PR and/or ERα hormone receptors status as a percentage of all FOXL2+ cells. Each vertical bar represents an individual image (*n* = 130 images), called ROI sorted by sample, grouped by primary (left) and recurrent (right) AGCT conditions. Colors correspond to subpopulations of FOXL2+ cells. **B–D,** t-SNE dimensionality reduction map representing subpopulations of FOXL2+ cells, with each color corresponding to a FOXL2+ cell subtype and each dot representing a single cell (**B**), t-SNE map overlaid with AGCT conditions (yellow, primary tumors; purple, recurrent tumors; **C**) and with the expression intensities of FOXL2, COL1A1, PR, ERα, calretinin, inhibinα, SF1, vimentin, αSMA, and Ki67 (**D**). **E** and **F,** Representative IMC images for COL1A1 (red), FOXL2 (blue), and PR (yellow) staining showing the difference between FOXL2+ cells in no-collagen and collagen-rich areas in PR expression and in cell shapes. Bean-shaped FOXL2+COL1A1− granulosa cells (**E** and **F**, orange arrows), spindle-shaped FOXL2+COL1A1+PR+ stromal elements (**E**, green arrows), and small round FOXL2+COL1A1+PR− stromal elements (**F**, white arrows). **G,** Characteristics of FOXL2+COL1A1−, FOXL2+COL1A1+, and stromal cells presented as a heatmap showing relative average expression of tumor and stromal markers across tumor and stromal cell subpopulations identified by IMC (*n* = 130 images). Actual markers are under the heatmap, phenotypic assignments are on the left, and the number of cells with related phenotype in the whole set of images is on the right of the heatmap. Heatmap color represents *Z*-score calculated per marker intensity. **H,** Box plots of comparison fractions of FOXL2+ cell subtypes significantly different between primary and recurrent AGCTs highlighting FOXL2+COL1A1+ cells subpopulations which significantly depleted in recurrent AGCTs. **I,** Comparison fractions of FOXL2+ cell subtypes and stromal cells between AGCT-1 and AGCT-2 highlighting more differences in cell fractions than in primary vs. recurrent AGCT comparison. **H** and **I,** Number of cells normalized per total cell number on relative image. Each dot represents the mean value of cell fraction from all ROIs in one sample. A Wilcoxon test was used to compare differences across conditions, with *P* values displayed on the plot.

Based on PR and ERα expression from IMC data, FOXL2+COL1A1− and FOXL2+COL1A1+ cells were classified on a per-cell basis as either PR+ERα+, PR+ERα−, PR−ERα+, and PR−ERα−. Among cells that were positive for at least one hormonal receptor, most were PR+ERα− (Fig. 3A), whereas PR+ERα+ cells and PR−ERα+ cells were uncommon. PR−ERα− cells were prevalent in most of the AGCT samples. A t-SNE analysis (Fig. 3B–D; Supplementary Fig. S11) showed distinguishable cellular clusters of FOXL2+ cells with distinct expression profiles, though not without some overlap. A t-SNE map of COL1A1 expression showed low overlap with PR expression, with expression of calretinin detected in the majority of FOXL2+ cells. Some FOXL2+ cells showed expression of αSMA and vimentin in both COL1A1− and COL1A1+ conditions (Fig. 3D). Interestingly, close examination of samples with FOXL2+COL1A1+ cells revealed that in some samples, PR was preferably expressed by FOXL2+ cells which are deeply embedded in collagen (Fig. 3E), whereas in other samples, PR was preferably expressed by FOXL2+ cells in regions depleted of COL1A1 (Fig. 3F).

To further explore the immunophenotype of FOXL2+ERα−PR− cell populations in AGCT, we next investigated the co-expression of other markers in our IMC panel. Specifically, in FOXL2+COL1A1− cells, we examined expression of other AGCT tumor markers such as SF1, calretinin, and inhibinα, whereas in FOXL2+COL1A1+ cells, we also examined expression of stromal markers, including αSMA, vimentin, S100A4, PDGFRa, PDGFRb, and FAP. We identified major FOXL2− cell populations that were αSMA+ and COL1A1+ and may represent CAFs (Fig. 3G). Based on this expanded analysis of IMC marker co-expression, FOXL2+COL1A1+ cells can be separated into two subgroups, one resembling FOXL2+COL1A1− tumor cells and the other with an immunophenotype resembling non-AGCT stromal cells. The first subgroup of FOXL2+COL1A1+ cells expressed other tumor markers (Fig. 3G, expression profiles highlighted by frames 1 and 2). Of note, those groups of FOXL2+COL1A1+ cells, besides positivity for COL1A1, had higher expression of αSMA, PDGFRa, and FAP (Fig. 3G, frame 2) than FOXL2+COL1A1− tumor cells themselves (Fig. 3G, frame 1). At the same time, the second subset of FOXL2+COL1A1+ did not express other AGCT markers besides FOXL2 and more closely resemble fibroblasts based on PDGFRa, PDGFRb, and S100A4 expression.

We next extended our analysis to compare fractions of those cell subpopulations between primary and recurrent AGCTs. The prevalence of FOXL2+COL1A1+ cells, which resemble stromal cells based on stromal marker expression and morphology, was significantly different between primary and recurrent AGCTs. Recurrent tumors demonstrated statistically significant depletion of several fractions of FOXL2+COL1A1+ cells (Fig. 3H). Interestingly, there was no difference in any other stromal cell subpopulations and no difference in subpopulations of FOXL2+COL1A1− cells (Supplementary Fig. S12). We observed statistically significant differences in abundance among specific cellular subpopulations between AGCT-1 and AGCT-2 (Fig. 3I). Besides the difference in fractions of FOXL2+COL1A1+ cells, we also detected differences in fractions of stromal cells (Fig. 3I, red frame, which again highlights more differences between identified subtypes rather than primary and recurrent conditions.

### Differences in immune cell composition across AGCT TME compartments

**Figure 4.**
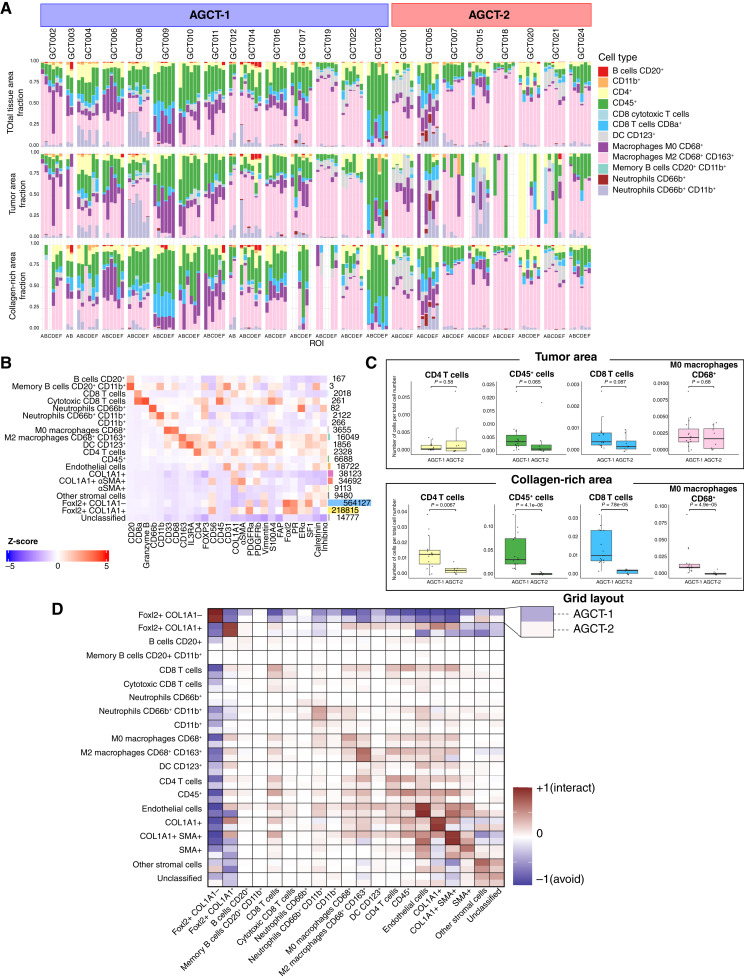
Immune cell composition differs across AGCT TME subtypes. **A,** The distribution of immune cell populations as a percentage of all immune cells in the AGCT TME. Each vertical bar represents an individual image (*n* = 130 images), called ROI sorted by sample, grouped by AGCT subtype: AGCT-1 (left) and AGCT-2 (right). Colors correspond to immune cell types. **B,** Heatmap showing relative average expression of all phenotypic markers across cell populations identified by IMC (*n* = 130 images). Actual markers are on the bottom, phenotypic assignments are on the left, and the number of cells with related phenotype in the whole set of images is on the right of the heatmap. Heatmap color represents *Z*-score calculated per marker intensity. **C,** Box plots indicating the proportion of detected immune cell types in AGCT-1 and AGCT-2 through tumor (top) and collagen-rich (bottom) areas. Number of immune cells normalized per total cell number in each area of relative image. Each dot represents the mean value of cell fraction from all ROIs in one sample. A Wilcoxon test was used to compare differences across primary and recurrent conditions, with *P* values displayed on the plot. **D,** Heatmap of pairwise cellular associations/interactions and avoidances between cell types (from cell type, *x*-axis; to cell type, *y*-axis). Cells are defined as interacting if the distance between centroids is <20 μm in distance. Interaction testing was performed with 1,000 iterations and assessed by comparing the number of interactions in each ROI with a null distribution of randomly permuted cell-type labels, with a significance threshold of 0.01. Only significant values were allowed to contribute, which averaged interaction–avoidance scores across ROIs: associations (red, +1) and avoidances (blue, −1) across samples per group. DC, dendritic cell.

We next examined the TME composition of immune cell subtypes in AGCT. For immune cell phenotyping, we used a supervised lineage assignment approach based on the expression of canonical immune cell markers (Supplementary Fig. S2) and identified 12 immune cell populations (Fig. 4A and B). Among immune cells, dominant cell populations included CD68^+^ and CD68^+^CD163^+^ macrophages, whereas lymphocytes were relatively less frequent (Fig. 4A). We did not detect FOXP3+ regulatory T cells and detected only a small number of CD20^+^CD11b^+^ memory B cells (only four cells per the whole set of samples). We compared the abundance of each immune cell population in primary and recurrent AGCT groups as well as in AGCT-1 and AGCT-2 subtypes. Considering the total tissue area, we observed a higher amount of macrophages M0 CD68^+^ in recurrent compared with primary AGCTs (*P* = 0.047; Supplementary Fig. S13), which is in concordance with our previous findings based on computational deconvolution from RNA-seq data (15) and data from immunofluorescence multiplexing assay analysis presented in a recent abstract (30).

Comparison of AGCT-1 and AGCT-2 showed higher abundance of undefined CD45^+^ immune cell populations (*P* = 0.00027; Supplementary Fig. S14) in AGCT-1, whereas other immune cell type populations did not differ significantly. Interestingly, when we compared immune cell abundance in compartments of pure tumor and collagen-rich areas separately, we observed that some immune cell subtypes were present in different proportions in those areas. Whereas we have not detected significant differences in CD8^+^ T cells and undefined CD45^+^ cell fractions in recurrent AGCTs in comparison with primary AGCTs in total tissue area, we observed significant increases of those cells in recurrent tumors in collagen-rich areas. Although it was not striking in primary versus recurrent comparison (Supplementary Fig. S13), we detected significant differences in immune cell abundance in collagen-rich areas between AGCT-1 and AGCT-2 subtypes (Fig. 4C; Supplementary Fig. S14). For example, CD4^+^ T cells, undefined CD45^+^, CD8^+^ T cells, and M0 macrophages CD68^+^ were much more abundant in the collagen-rich area of AGCT-1 in comparison with AGCT-2, whereas immune cell composition within the tumor area was not significantly different.

We next expanded on these analyses to investigate immune cell colocalization within the AGCT TME. We first examined patterns of cellular interaction and avoidance by applying a permutation test to infer colocalization and identify significant deviations from a random cellular arrangement for each ROI. Specifically, this analysis reveals the tendency of each pair of cell types to directly interact beyond random chance. For simplicity of data interpretation for this analysis, we used immune cell subpopulations with other major key cell populations without subdividing FOXL2+COL1A1−, FOXL2+COL1A1+, and stromal cells into subpopulations.

As anticipated, tumor cells and endothelial cells tended to colocalize with cells of the same type (Fig. 4D; Supplementary Fig. S15). COL1A1+ stromal cells tended to colocalize with one another as well. Across all interactions, the most frequent nonself interaction occurred between endothelial cells and COL1A1+αSMA+ cells, as well as between endothelial cells and αSMA+ cells. We noted that endothelial cells, CD8 T cells, M2 macrophages, COL1A1+ cells, and COL1A1+αSMA+ cells were most strongly avoidant of FOXL2+COL1A1− tumor cells. Among immune cells, M2 macrophages, neutrophils CD66b+CD11b+, and CD8 T cells preferred to interact with the same cell type relatively more frequently than with other cell types.

Comparing cell interaction profiles between AGCT-1 and AGCT-2 subtypes, we observed overall more frequent interactions in AGCT-1 between CD8 T cells, M0 and M2 macrophages, CD4^+^ T cells, CD45^+^ cells, endothelial cells, COL1A1+ cells, and COL1A1+αSMA+ cells (Fig. 4D). At the same time FOXL2+COL1A1− tumor cells in AGCT-1 avoid other cells more frequently than in AGCT-2 (Fig. 4D), which can suggest less infiltration level of any other cells in AGCT-1 samples. A nearest neighbor analysis demonstrated more interactions between CD8 T cells, M0 and M2 macrophages, and CD45^+^ cells with endothelial cells in AGCT-1 compared with AGCT-2, highlighting the much closer proximity of those cells to blood vessels in the TME. Comparison of primary and recurrent AGCTs also showed more interactions between CD8 T cells, M0 and M2 macrophages, CD4^+^ T cells, CD45^+^ cells, endothelial cells, COL1A1+ cells, and COL1A1+αSMA+ cells in recurrent tumors, whereas closer association of CD8 T cells, M0 and M2 macrophages, and CD45^+^ cells with endothelial cells and blood vessels was not so striking between primary and recurrent tumors. FOXL2+COL1A1− tumor cells in recurrent samples avoided other cells more frequently than in primary AGCTs (Supplementary Fig. S15).

### AGCT TME subtypes have distinct transcriptomic profiles

**Figure 5.**
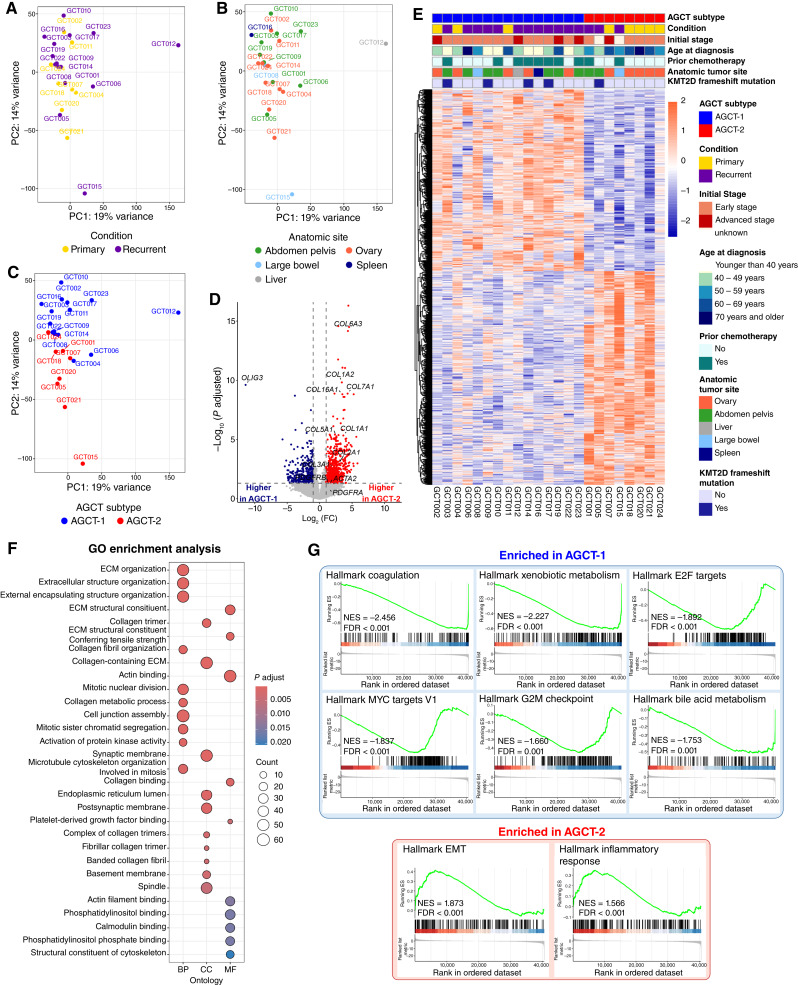
AGCT TME subtypes have distinct gene expression programs. **A,** PCA based on bulk RNA-seq data highlighting minimal transcriptomic differences between primary and recurrent AGCTs. **B** and **C,** PCA plot overlaid with anatomic tumor site (**B**) and AGCT IMC subtypes (**C**). **D,** Volcano plot of DEGs between AGCT subtypes. The *x*-axis indicates the FC (log_2_ scaled), and the *y*-axis shows the adjusted *P* value (log_10_ scaled). Each point represents a different gene; the red/blue color of the points categorize genes with increased/decreased expression in the AGCT-2 subtype, highlighting collagen-related genes as top genes with increased expression in AGCT-2. **E,** Differential gene expression analysis results presented as an expression heatmap of all DEGs in which rows represent genes and columns represent AGCT samples. Clinicopathologic annotations presented at the top of the heatmap. The variance stabilizing transformation was applied for expression data. Colors of heatmap display gene expression *Z*-scores that are computed on a gene-by-gene basis: Increased expression (red) and decreased expression (blue). **F,** Results of the functional enrichment analysis performed with DEGs and GO terms showing that primarily ECM and collagen-related GO terms are altered between AGCT subtypes. Top 10 GO terms for every category are presented. **G,** Top significantly enriched gene sets from MSigDB hallmark gene sets revealed by GSEA. BP, biological process; CC, cellular component; ES, enrichment score; MF, molecular function; NES, normalized enrichment score.

To gain insights into gene expression programs that may underlie the AGCT-1 and AGCT-2 TME subtypes, we next integrated our IMC data with our previously published bulk RNA-seq data from these samples (15). Previously, by performing differential gene expression analysis and comparing primary and recurrent AGCT samples, we identified only small transcriptomic differences and a small number of DEGs (15), highlighting that transcriptional profiles between primary and recurrent tumors do not differ significantly. This minimal difference is reflected in the PCA plot (Fig. 5A) generated from the RNA-seq data. Samples from different anatomic sites also intermixed on the PCA plot (Fig. 5B), highlighting that whereas site differences can influence cellular composition, particularly stromal composition, our findings suggest that the IMC-based TME subtype, rather than anatomical site, plays a more dominant role in shaping both stromal composition and the observed molecular differences. Interestingly, differences in fractions of FOXL2+COL1A1+ cells, which were used as main criteria for AGCT TME subtype division, also reflected on PCA colored by AGCT-1 and AGCT-2 subtypes (Fig. 5C) and indeed indicate the presence of different transcriptional profiles.

Differential gene expression analysis between AGCT-1 and AGCT-2 subtypes identified a total of 1,275 DEGs for which expression was significantly different between the two studied groups (adjusted *P* < 0.05, FC >2). At the gene expression level, among the top DEGs between AGCT-1 and AGCT-2 were genes coding different collagens, including COL1A1 (adjusted *P* = 0.00197; log_2_ FC = 2.83; Fig. 5D) with higher expression in AGCT-2 subtype. The full list of analyzed genes and results of differential gene expression analysis between AGCT-2 and AGCT-1 with *P* values and FCs, calculated by DESeq2 are provided as Supplementary Table S2. At the genetic level, truncating KMT2D mutations were more common in AGCT-1 (4/15; 26.7%) compared with AGCT-2 (1/8; 12.5%; Fig. 5E).

To better understand the differences in AGCT subtypes and functional role of identified DEGs, we performed enrichment analysis for GO terms (Fig. 5F). Among the top enriched GO terms were GO terms related to ECM and collagen organization, highlighting collagen remodeling as the most important feature between AGCT TME subtypes and suggesting that tumors from different subtypes can behave differently because of differences in collagen properties. The full list of enriched GO terms between AGCT-2 and AGCT-1 is provided as Supplementary Table S3. GSEA of hallmark gene sets from the Molecular Signatures Database revealed the next top gene sets enriched in AGCT-1: coagulation, xenobiotic and bile acid metabolism, E2F and MYC targets, and G2M checkpoint gene sets (Fig. 5G; Supplementary Table S4). Epithelial–mesenchymal transition (EMT) and inflammatory response were the top-enriched gene sets in AGCT-2 (Fig. 5G). This suggests that AGCT-2 may reflect a TME subtype with increased propensity for EMT.

## Discussion

The main purpose of this study was to explore AGCTs’ spatially resolved TME ecosystems, identify TME differences between primary and recurrent tumors that may correlate with recurrence, and establish novel biomarkers associated with recurrence of AGCTs that may allow for identification of potential therapeutic targets. To do this, we applied the IMC approach to 24 FFPE tumor tissue samples using a custom designed IMC panel with 34-markers related to AGCT biology.

To study the TME composition, it is useful to distinguish tumor from stromal regions. However, in the present study, due to the big overlap of FOXL2 and COL1A1 staining, we could not make a clear separation between tumor and stromal areas. Instead, we highlighted pure tumor and collagen-rich areas. We recognize that fibromatous tumors without distinguishable stroma may exhibit distinct behavior compared with collagen-rich stromal regions from other samples with a distinguishable tumor–stroma interface. However, for this study, we classified all collagen-rich areas as a single histologic category, reasoning that COL1A1+ fibromatous tumors may exhibit behavior more similar to stromal areas with FOXL2+ cells of any kind than to pure tumor regions without collagen. Having a bigger cohort could allow us to consider fibromatous tumors separately from samples with distinguishable tumor and stromal regions, which was not an option in the current study.

Comparison of the TME cell composition between primary and recurrent AGCTs revealed a relatively small number of cell populations that differ significantly between two conditions. What caught our attention is that the fractions of several subtypes of FOXL2+COL1A1+ cells were significantly depleted in recurrent AGCTs. Interestingly, there was no difference in any other stromal cell subpopulations and in subpopulations of FOXL2+COL1A1− cells (Supplementary Fig. S12). In our previous work, computational deconvolution of bulk RNA-seq data suggested a depletion of CAFs in recurrent tumors (15). In contrast, in the present study, the abundance of classic CAFs did not differ significantly between primary and recurrent tumors. This difference may be because deconvolution methods misidentified FOXL2+COL1A1+ cells as CAFs, which could explain the reported CAF depletion in recurrent tumors.

In the present work, we highlight that FOXL2+COL1A1+ cells are FOXL2+ cells with nontypical “coffee bean–shaped” morphology, which are deeply embedded in COL1A1+ areas. Based on the patterns of expression αSMA, FAP, and other CAF markers in those cells, we do not exclude the possibility that some of those FOXL2+COL1A1+ cells can be stromal cells and can express COL1A1. This is consistent with previous reports that FOXL2+ cells can differentiate into both granulosa and ovarian stromal cell lineages, which express COL1A1 (31). Also, previously FOXL2 expression has been observed in fibroblast-like stromal cells of ovarian cancers (32) and ovarian-type stroma in nonovarian tumors (33). In this study, in the case of AGCT, we highlight that the expression of FOXL2 is not restricted to the stroma of ovaries; in fact, FOXL2+ stromal cells were also identified in other organs examined.

We suppose that presence of FOXL2+ stromal cells in AGCTs may play a pivotal role in tumor progression and treatment response. The presence of those cells in collagen-rich areas also correlated with higher expression of hormonal receptors in stromal areas, suggesting distinct properties to the stroma of those tumors and a potential role of FOXL2+ stromal cells in modulating hormone responsiveness and ECM dynamics. In breast cancer, ER and PR expression has been associated with ECM remodeling in TME, cancer progression, and treatment response (34–37). In endometrial cancer, stromal PR was found to influence the response to hormonal therapy (38). Future studies could explore whether ER/PR signaling in FOXL2+COL1A1+ cells modulates ECM composition and mechanical properties in AGCTs, potentially influencing tumor recurrence or therapy response.

Also, current work confirmed an increase in M0 CD68^+^ macrophages in recurrent tumors in comparison with primary tumors, especially in collagen-rich areas. In addition, recurrent tumors showed higher fractions of CD8^+^ T cells and CD45^+^ cells compared with collagen-rich areas of primary tumors. Taken together, these results provide insights into the TME composition of AGCT samples and suggest a role for both macrophages and FOXL2+ cells in collagen-rich areas in shaping AGCT recurrence.

Whereas we have built our prior work (15) and a part of our current work examining differences between primary and recurrent AGCTs, here we report the first comprehensive spatial analysis of the AGCT TME. Whereas the TME of primary and recurrent AGCTs are frequently similar in many respects, distinct AGCT TME subtypes exist that are defined by the close association between FOXL2+ tumor cells and local COL1A1. We propose two AGCT TME subtypes as the COL1A1-depleted AGCT-1 subtype and COL1A1-intermixed AGCT-2 subtype. Though FOXL2+COL1A1+ cell abundance guided subtype assignment, we note that these cells showed heterogeneous morphologies across samples. Further subdivision by cell morphology, for example, or other histologic features can also be useful as different cell morphologies can reflect different molecular profiles and cell functions. We speculate that spindle-shaped FOXL2+COL1A1+ cells may behave more like fibroblasts and contribute to collagen synthesis, deposition, and remodeling of the ECM, whereas small round FOXL2+COL1A1+ cells may represent tumor cells undergoing EMT.

Another potential way to look at AGCT is to investigate ECM and collagen characteristics. Visual inspection of IMC images with COL1A1 and tumor marker staining pointed out the presence of diverse collagen structures, different levels of collagen fiber alignment, and different levels of tumor–stroma demarcation from perfect segregation to completely mixed areas. Recurrent tumors seemed to have less COL1A1+ area, consistent with bulk RNA-seq data from our earlier study (15), which showed lower level of COL1A1 in recurrent AGCTs (log_2_ FC = 2.39; *P* = 0.0016; adjusted *P* = 0.24). Moreover, the collagen-containing ECM GO term gene set was one of the top enriched cellular components in the same previous study (15), reinforcing collagen’s potential role in shaping AGCT recurrence.

Comparing collagen parameters between AGCT subtypes, we observed a large difference in collagen area presence between AGCT-1 and AGCT-2, with higher collagen proportion in AGCT-2 and different cell density in the collagen-rich area. Comparative transcriptomic analysis also highlighted ECM-related differentially enriched GO terms between AGCT-1 and AGCT-2. Although we did not have enough data to perform survival analysis between AGCT subtypes, we think that a study of larger AGCT cohorts with different collagen presence and a study of collagen features can lead to insights about aggressiveness and treatment response of this type of tumor. Previously, using collagen features in other cancer types showed usefulness in survival prediction (39), but in AGCT, it has not been studied. Collagen presence can provide mechanical restriction for tumor progression, and collagen depletion in those cases can lead to lower tissue stiffness, accelerated tumor growth, and diminished overall survival (40, 41). Although we have not studied collagen features more deeply, our findings suggest that AGCT subtypes based on ECM features may better inform treatment planning than targeting a few DEGs such as IDO1, SPP1, and VEGFA (30), which were not identified as DEGs in our AGCT cohort (Supplementary Fig. S16; ref. 15).

### Strengths and limitations of this study

A significant strength of this study is the use of a relatively large and clinically well-characterized cohort for a rare cancer. Diagnoses were confirmed not only by H&E but also by the presence of FOXL2 staining and AGCT-specific somatic FOXL2 c.C402G mutation in 23 of 24 cases (10).

Another key strength is the use of a 34-marker IMC panel specifically designed to capture key aspects of AGCT biology, enabling deep phenotyping and cell identity assignment for 98% of cells. Because IMC is imaging-based, it not only provides spatial context but also allows for the direct observation of tissue architecture and cell morphology. This visual context led to the identification of two distinct AGCT subtypes with greater transcriptional differences than the primary versus recurrent classification, suggesting that TME-driven subtyping, informed by imaging, may better capture biologically and clinically meaningful heterogeneity in AGCT. IMC also revealed distinct morphologic features of FOXL2+ cells, particularly in collagen-rich regions, which would be difficult to access using non–imaging-based single-cell techniques. Additionally, the spatial resolution of IMC allowed for the mapping of cell-to-cell interactions, offering unique insights into AGCT TME organization. Together, these strengths enhance the depth and translational relevance of our findings, setting a foundation for the future integration of imaging-based subtyping into precision oncology strategies for AGCT.

Among the study’s limitations, we highlight the use of ROIs rather than full tissue sections. Using the whole tumor tissue sample slide could provide deeper insights into AGCT TME composition. Another common limitation of spatial imaging technologies is the analysis of 3D cells in 2D tissue sections, which affects observed morphology depending on the plane of sectioning. Therefore, there was a limitation in fully evaluating the cell morphology even under high-resolution images. This may be resolved by further development of 3D spatial imaging technologies.

It also needs to be highlighted that microscopic intratumoral hemorrhage is a common aspect of AGCTs. Previously, it was shown that hemorrhage-activated processes in tumor-associated macrophages can drive cancer growth, invasion, and immunotherapy resistance (42), yet in our work, it was largely ignored. On the ROI selection step, we tended to select tumor–stromal interfaces, whereas hemorrhagic areas were excluded and not analyzed.

Prior treatment history could also significantly affect the TME; however, due to the small sample size in combination with the diversity of treatment procedures and regimens, we did not broadly discuss treatment history in the current article, which can also be considered a limitation of this study.

Finally, a limitation of our study is the use of nonmatched AGCT samples due to the unavailability of high-quality paired primary and recurrent tissues. Despite extensive review of clinical and pathology archives, only three cases with sequential tumor blocks were found, most being too old or of insufficient quality for IMC. This reflects the referral nature of our institution, in which recurrent cases often present years after initial treatment elsewhere. Whereas paired analysis would offer valuable insights, the small sample size and quality limitations restrict feasibility. Nonetheless, matched samples could better reveal TME evolution, including age-related changes and potential receptor or subtype switching, which may affect treatment planning, as suggested in breast cancer (43, 44). If AGCT subtypes correlate with tumor progression and survival, study of bigger cohorts with paired samples can be interesting in terms of this subtype or receptor status switching in AGCTs.

## Supplementary Material

Supplementary Table S1 Table S1. Antibodies and Metals/Isotopes Used for Imaging Mass Cytometry.

Supplementary Table S2 Supplementary Table S2. Schematic for cell lineage assignment strategy.

Supplementary Table S3 Supplementary Table S3. Enriched GO terms between AGCT-2 and AGCT-1.

Supplementary Table S4 Supplementary Table S4. GSEA results with enriched hallmark gene sets between AGCT-2 and AGCT-1.

Supplementary Figure S1 Figure S1. Representative staining of antibody and nuclear markers used for imaging mass cytometry.

Supplementary Figure S2 Figure S2. Lineage assignment strategy

Supplementary Figure S3 Figure S3. Fractions of major cell types detected in primary and recurrent AGCT samples

Supplementary Figure S4 Figure S4. t-SNE dimensionality reduction map for major cell types in AGCT TME

Supplementary Figure S5 Figure S5. Foxl2+ cells with different cell morphology

Supplementary Figure S6 Figure S6. Density plot of Foxl2+COL1A1+ cell proportions showing a bimodal distribution

Supplementary Figure S7 Figure S7. Principal component analysis plot representing AGCT samples colored by anatomical tumor site

Supplementary Figure S8 Figure S8. COL1A1 area and expression intensity in primary vs recurrent AGCT tissue samples

Supplementary Figure S9 Figure S9. PR and ER expression intensity in primary vs recurrent AGCT tissue samples

Supplementary Figure S10 Figure S10. IMC and IHC staining comparison: PR staining

Supplementary Figure S11 Figure S11. t-SNE dimensionality reduction map for Foxl2+ cells in AGCT TME

Supplementary Figure S12 Figure S12. Differences in fractions of Foxl2+ COL1A1-, Foxl2+ COL1A1+, and stromal cells

Supplementary Figure S13 Figure S13. Differences in fractions of immune cells in different tissue compartments of primary and recurrent AGCTs

Supplementary Figure S14 Figure S14. Differences in fractions of immune cells in different tissue compartments of AGCT subtypes

Supplementary Figure S15 Figure S15. Cell-cell interaction comparative analysis

Supplementary Figure S16 Figure S16. Expression of IDO1, SPP1, and VEGFA genes in primary vs recurrent AGCT samples

## Data Availability

RNA-seq data for studied AGCT samples are available through the EGA under accession EGAS00001006478. The source data supporting the findings of this study, including high-dimensional IMC OME-TIFF images, H&E images, images mapping each ROI to the corresponding adjacent H&E, and clinical information, including primary/recurrent condition status, age, tumor stage and tumor size at diagnosis, anatomic tumor site, treatment history, and menopause status, have been deposited at https://doi.org/10.5281/zenodo.15384724. Code used for processing and analysis is available upon request.
