# Supplementary material for: Multiplexed Imaging Mass Cytometry Reveals Tumor-immune Microenvironment–dependent Hormone Receptor Expression in Adult-Type Ovarian Granulosa Cell Tumors
Source: Cancer Res Commun. 2025 Oct 27;5(10):1894–909. doi: 10.1158/2767-9764.CRC-25-0333 (PMC12555029; doi:10.1158/2767-9764.CRC-25-0333)
Supplement: Supplementary Figure S1 — Figure S1. Representative staining of antibody and nuclear markers used for imaging mass cytometry. [file crc-25-0333_supplementary_figure_s1_suppsf1.pdf]

Supplementary Figure S1. Representative staining of antibody and nuclear markers used for imaging mass cytometry.

A.

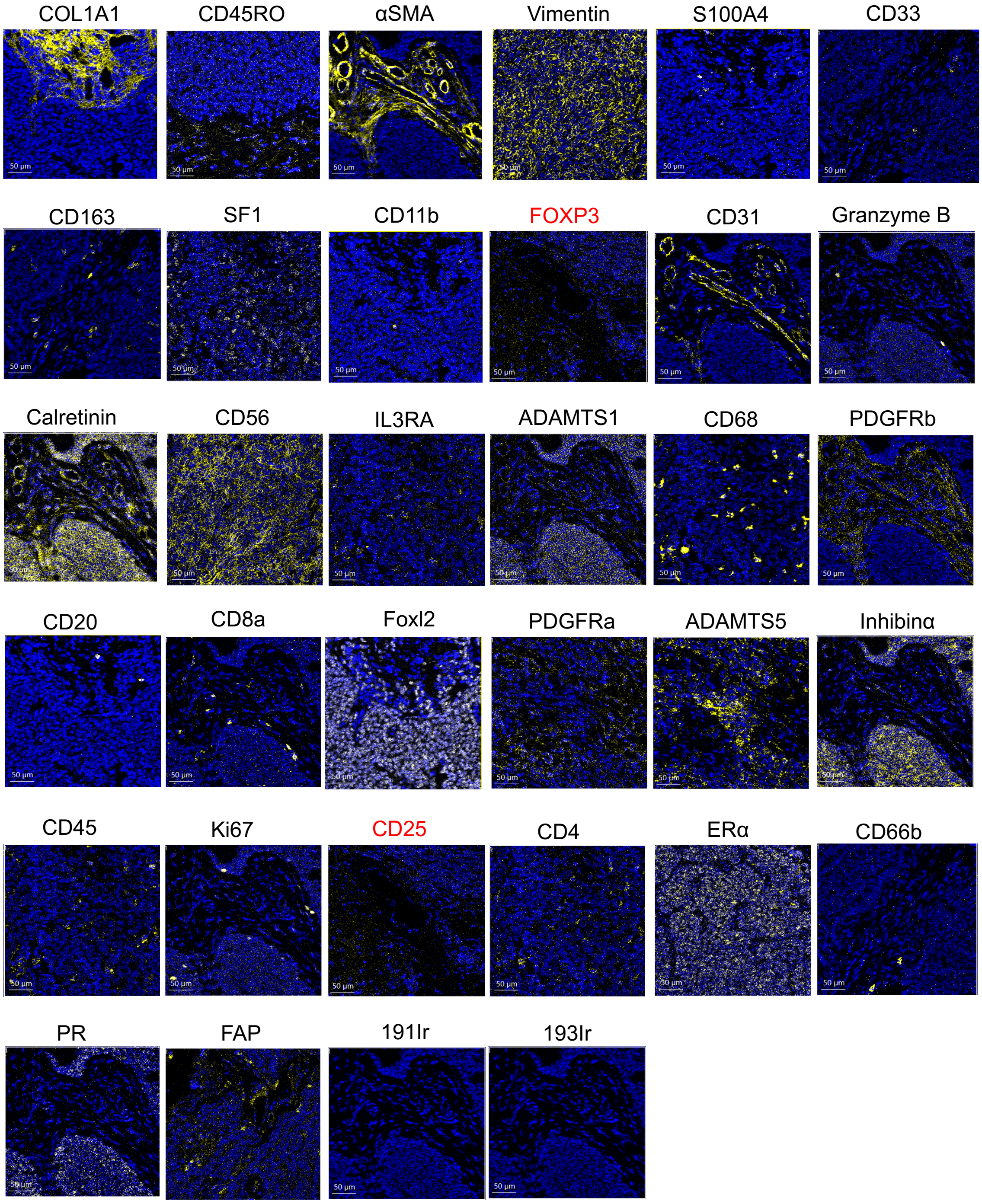

B.

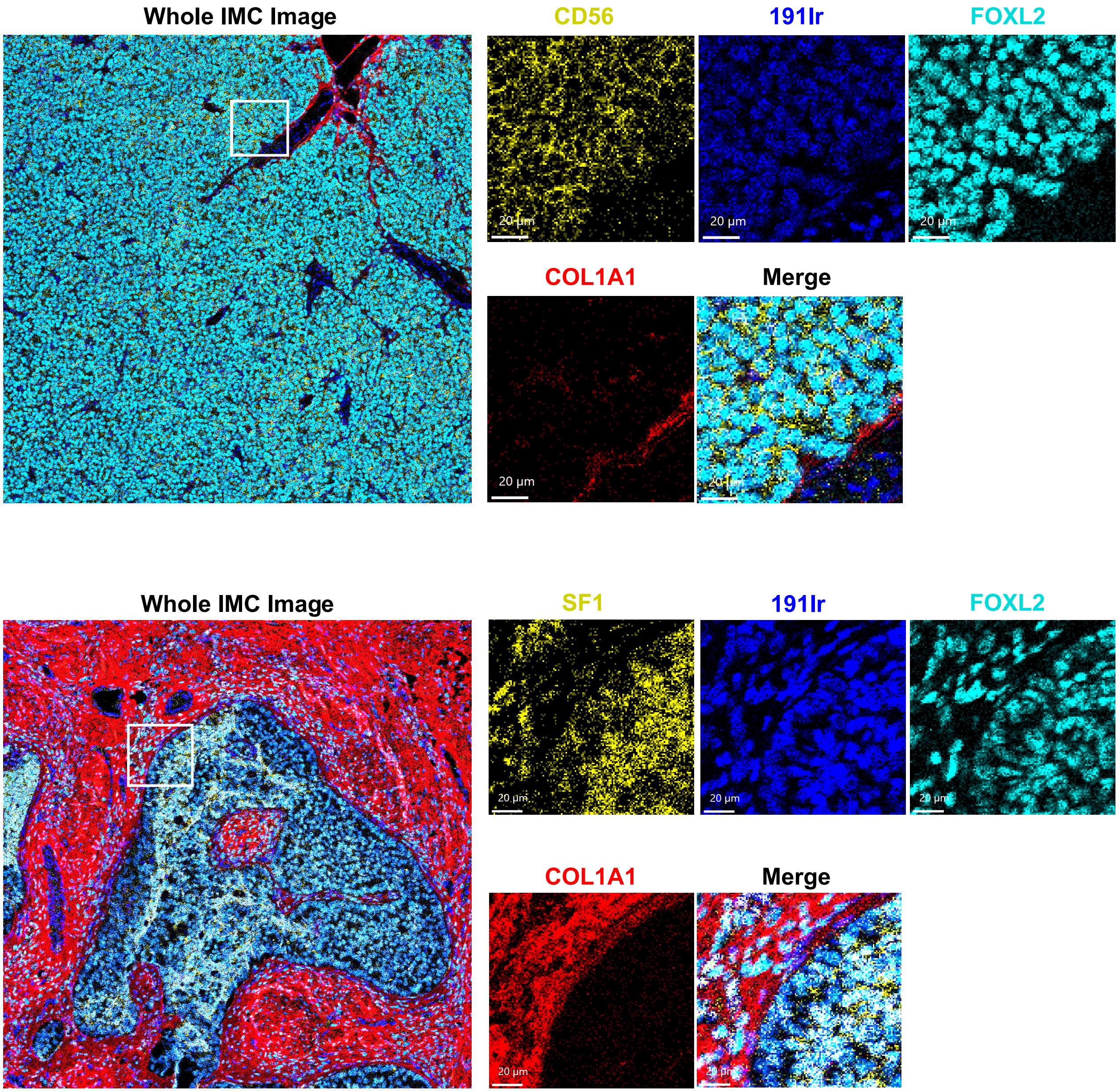
Whole IMC Image
CD56
191Ir
FOXL2
20 µm
20 µm
20 µm
COL1A1
Merge
20 µm
Whole IMC Image
SF1
191Ir
FOXL2
20 µm
20 µm
20 µm
COL1A1
Merge
20 µm

B. (continued)

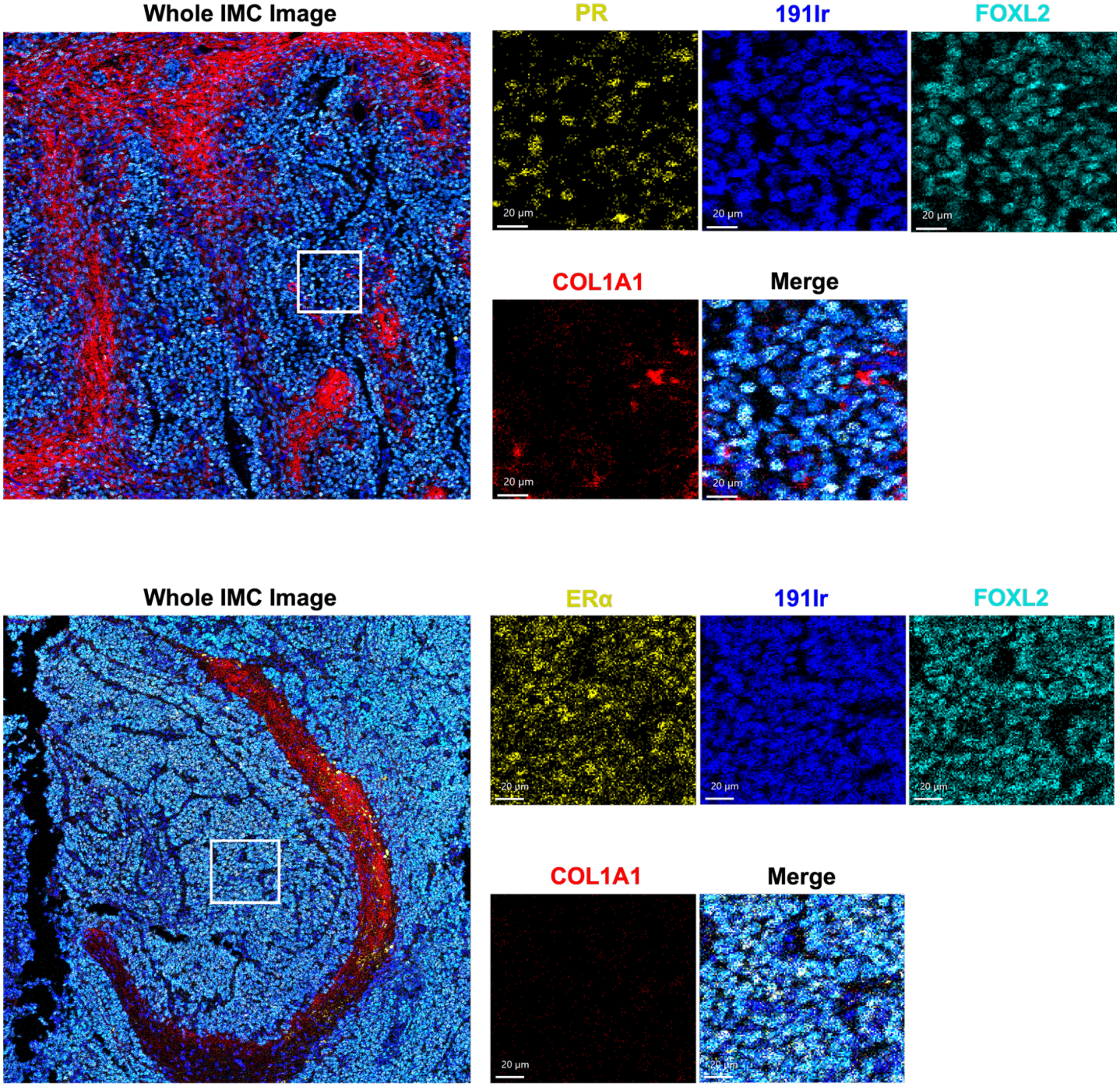

Supplementary Figure S1. A. *Representative IMC images for each marker included in the IMC panel. Images for different antibodies may correspond to different tissue samples. Nuclear labeling (blue) corresponds to Cell-ID Intercalator-Ir (191Ir or 193Ir), respective markers are shown in yellow. Marker names shown in red indicate antibodies that were detected in AGCT samples during the antibody validation step but were not detected in the analyzed cohort.* ***B****. Representative IMC images for CD56, SF1, PR or ERα (yellow), 191Ir (blue), FOXL2 (cyan), and COL1A1 (red) staining, highlighting consistent staining with expected patterns of morphology and subcellular localization for FOXL2, CD56, SF1, PR, Erα, COL1A1, and 191Ir.*
