# Supplementary material for: Multiplexed Imaging Mass Cytometry Reveals Tumor-immune Microenvironment–dependent Hormone Receptor Expression in Adult-Type Ovarian Granulosa Cell Tumors
Source: Cancer Res Commun. 2025 Oct 27;5(10):1894–909. doi: 10.1158/2767-9764.CRC-25-0333 (PMC12555029; doi:10.1158/2767-9764.CRC-25-0333)
Supplement: Supplementary Figure S2 — Figure S2. Lineage assignment strategy [file crc-25-0333_supplementary_figure_s2_suppsf2.pdf]

Supplementary Figure S2. Lineage assignment strategy

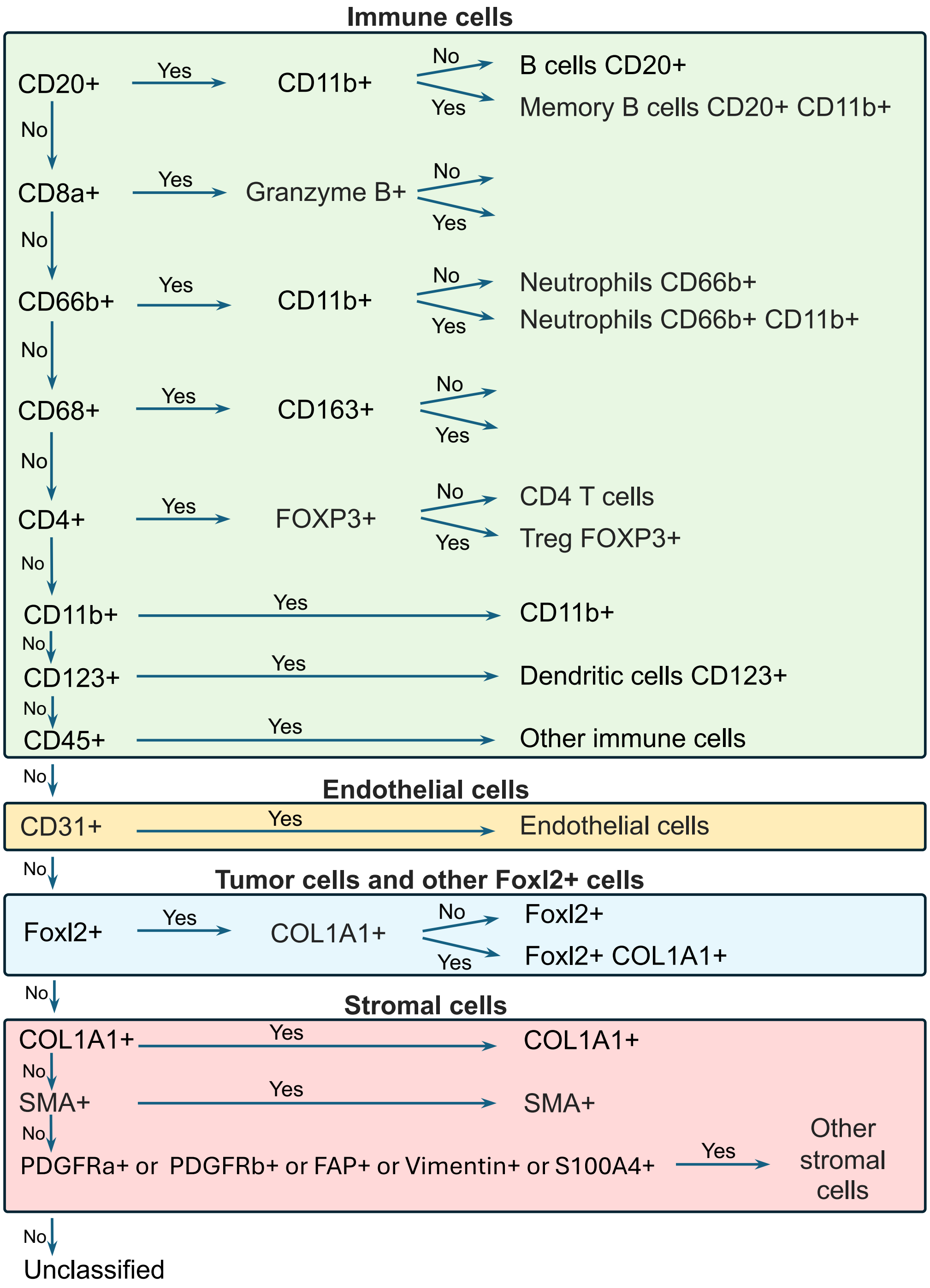

Supplementary Figure S2. *Schematic for cell lineage assignment strategy.*
