# Supplementary material for: Multiplexed Imaging Mass Cytometry Reveals Tumor-immune Microenvironment–dependent Hormone Receptor Expression in Adult-Type Ovarian Granulosa Cell Tumors
Source: Cancer Res Commun. 2025 Oct 27;5(10):1894–909. doi: 10.1158/2767-9764.CRC-25-0333 (PMC12555029; doi:10.1158/2767-9764.CRC-25-0333)
Supplement: Supplementary Figure S3 — Figure S3. Fractions of major cell types detected in primary and recurrent AGCT samples [file crc-25-0333_supplementary_figure_s3_suppsf3.pdf]

Supplementary Figure S3. Fractions of major cell types detected in primary and recurrent AGCT samples

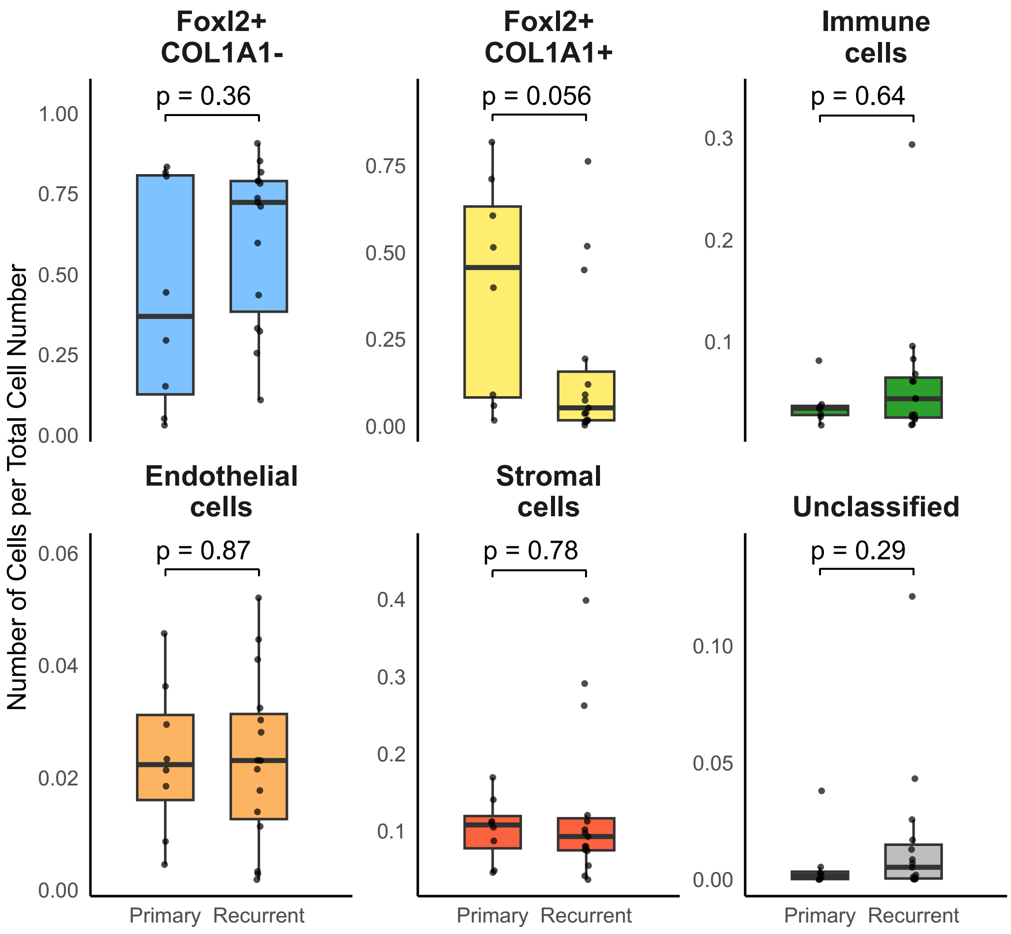

Supplementary Figure S3. *Comparison fractions of major cell types detected in primary and recurrent AGCT samples. Number of cells normalized per total cell number on relative image. Each dot represents the mean value of cell fraction from all ROIs in one sample. A Wilcoxon test was used to compare differences across primary and recurrent conditions, with p-values displayed on the plot.*
