# Supplementary material for: Multiplexed Imaging Mass Cytometry Reveals Tumor-immune Microenvironment–dependent Hormone Receptor Expression in Adult-Type Ovarian Granulosa Cell Tumors
Source: Cancer Res Commun. 2025 Oct 27;5(10):1894–909. doi: 10.1158/2767-9764.CRC-25-0333 (PMC12555029; doi:10.1158/2767-9764.CRC-25-0333)
Supplement: Supplementary Figure S4 — Figure S4. t-SNE dimensionality reduction map for major cell types in AGCT TME [file crc-25-0333_supplementary_figure_s4_suppsf4.pdf]

Supplementary Figure S4. t-SNE dimensionality reduction map for major cell types in AGCT TME

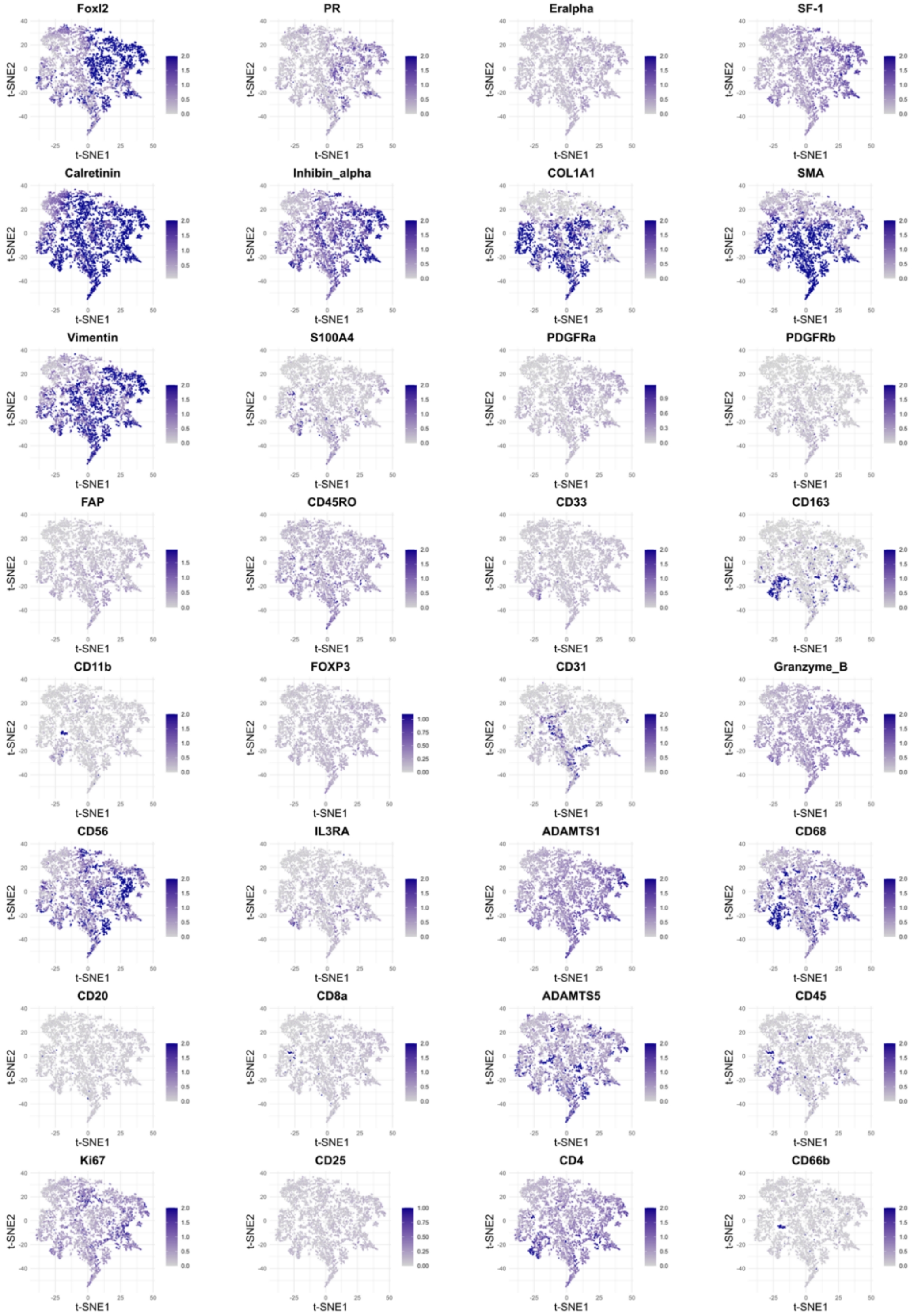

Supplementary Figure S4*. t-SNE dimensionality reduction map representing the expression intensities of all markers included in the AGCT IMC panel. Each dot represents a single cell. For the t-SNE analysis, 1,000 cells were randomly selected from each major cell group, totaling 6,000 cells.*
