# Supplementary material for: Multiplexed Imaging Mass Cytometry Reveals Tumor-immune Microenvironment–dependent Hormone Receptor Expression in Adult-Type Ovarian Granulosa Cell Tumors
Source: Cancer Res Commun. 2025 Oct 27;5(10):1894–909. doi: 10.1158/2767-9764.CRC-25-0333 (PMC12555029; doi:10.1158/2767-9764.CRC-25-0333)
Supplement: Supplementary Figure S5 — Figure S5. Foxl2+ cells with different cell morphology [file crc-25-0333_supplementary_figure_s5_suppsf5.pdf]

Supplementary Figure S5. Foxl2+ cells with different cell morphology

A.

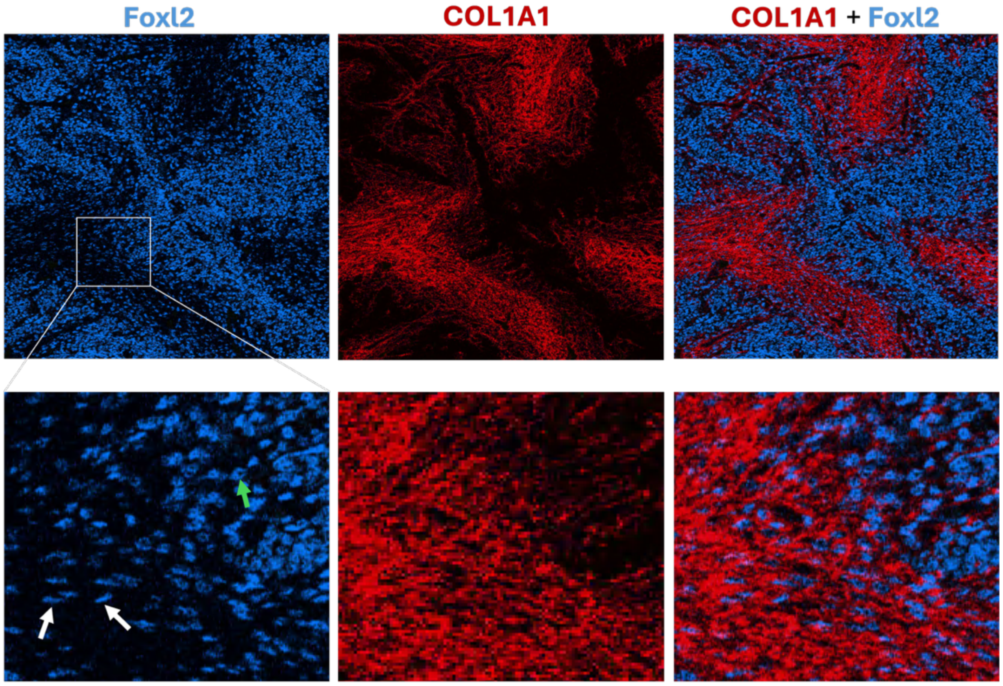

B.

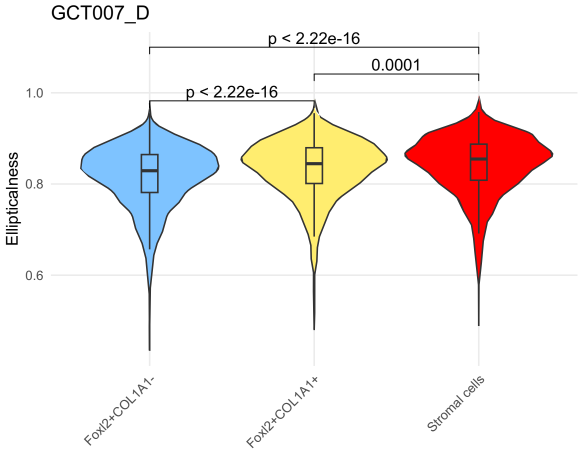

Supplementary Figure S5. A. *Representative IMC images from an AGCT sample showing FOXL2+ cells with distinct morphologies: classic "coffee bean-shaped" AGCT cells (green arrow) and FOXL2+ cells with irregular or spindle-shaped morphology located within COL1A1+ regions (white arrows).* ***B.*** *The latter population of FOXL2+COL1A1+ cells appeared more elongated (with a higher ellipticalness parameter) and resembled stromal elements such as cancer-associated fibroblasts, in contrast to the typical morphology of classic FOXL2+COL1A1- AGCT cells.*
