# Supplementary material for: Multiplexed Imaging Mass Cytometry Reveals Tumor-immune Microenvironment–dependent Hormone Receptor Expression in Adult-Type Ovarian Granulosa Cell Tumors
Source: Cancer Res Commun. 2025 Oct 27;5(10):1894–909. doi: 10.1158/2767-9764.CRC-25-0333 (PMC12555029; doi:10.1158/2767-9764.CRC-25-0333)
Supplement: Supplementary Figure S6 — Figure S6. Density plot of Foxl2+COL1A1+ cell proportions showing a bimodal distribution [file crc-25-0333_supplementary_figure_s6_suppsf6.pdf]

Supplementary Figure S6. Density plot of *Foxl2+COL1A1+* cell proportions showing a bimodal distribution

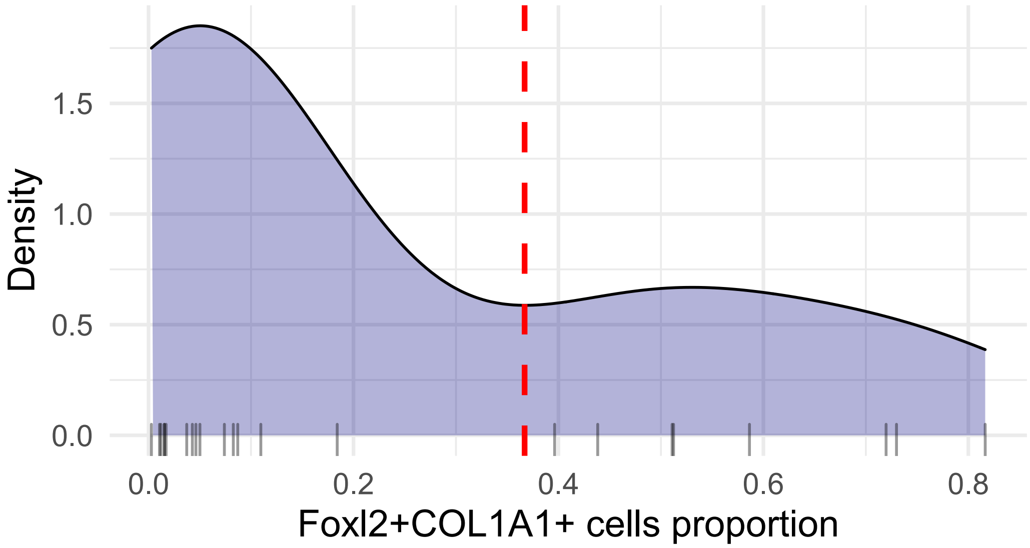

Supplementary Figure S6. *Distribution of Foxl2+COL1A1+ cell proportions across all samples. The density curve shows a bimodal distribution, indicating the presence of two distinct subgroups. The red dashed vertical line marks the threshold separating these subgroups, defined using the valley method (local minimum of the density curve between the two peaks). Black tick marks along the x-axis indicate individual sample values.*
