# Supplementary material for: Multiplexed Imaging Mass Cytometry Reveals Tumor-immune Microenvironment–dependent Hormone Receptor Expression in Adult-Type Ovarian Granulosa Cell Tumors
Source: Cancer Res Commun. 2025 Oct 27;5(10):1894–909. doi: 10.1158/2767-9764.CRC-25-0333 (PMC12555029; doi:10.1158/2767-9764.CRC-25-0333)
Supplement: Supplementary Figure S7 — Figure S7. Principal component analysis plot representing AGCT samples colored by anatomical tumor site [file crc-25-0333_supplementary_figure_s7_suppsf7.pdf]

Supplementary Figure S7. Principal component analysis plot representing AGCT samples colored by anatomical tumor site

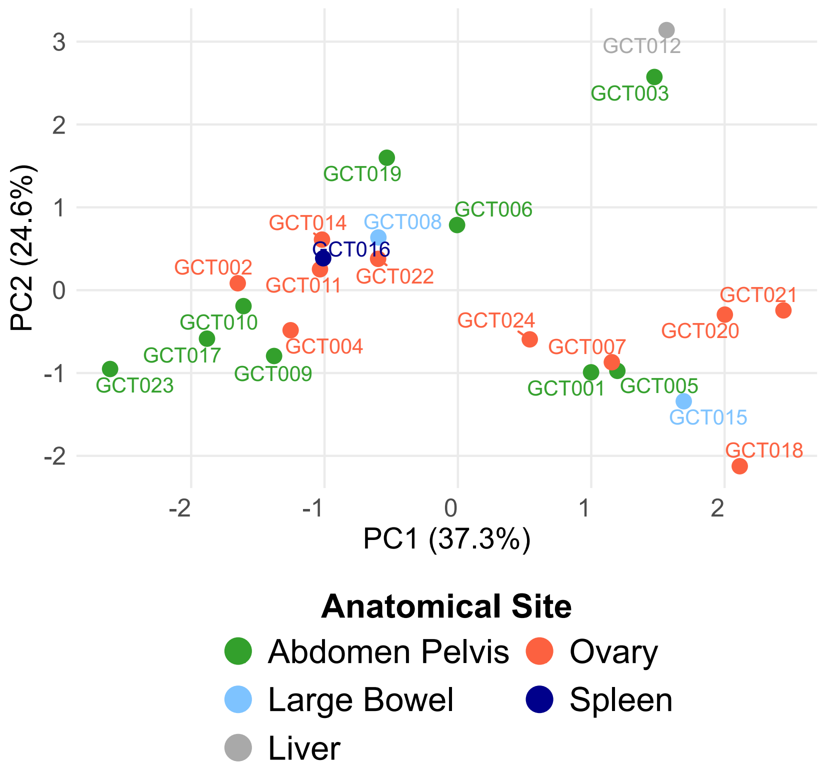

Supplementary Figure S7. *Principal component analysis plot representing the relationships among AGCT samples based on the composition of major cell types within the AGCT tumor microenvironment. Colors indicate AGCT samples derived from different anatomical collection sites. The clustering pattern suggests minimal differences in the composition of major cell types between tumor sites.*
