# Supplementary material for: Multiplexed Imaging Mass Cytometry Reveals Tumor-immune Microenvironment–dependent Hormone Receptor Expression in Adult-Type Ovarian Granulosa Cell Tumors
Source: Cancer Res Commun. 2025 Oct 27;5(10):1894–909. doi: 10.1158/2767-9764.CRC-25-0333 (PMC12555029; doi:10.1158/2767-9764.CRC-25-0333)
Supplement: Supplementary Figure S8 — Figure S8. COL1A1 area and expression intensity in primary vs recurrent AGCT tissue samples [file crc-25-0333_supplementary_figure_s8_suppsf8.pdf]

Supplementary Figure S8. COL1A1 area and expression intensity in primary vs recurrent AGCT tissue samples

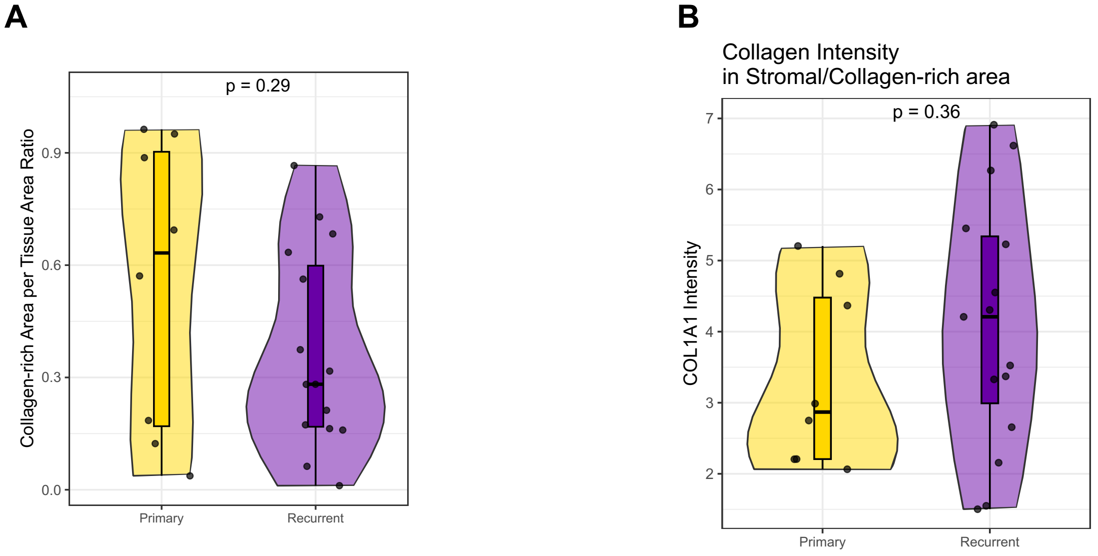

Supplementary Figure S8. *Overview of the collagen presence in primary vs recurrent AGCT tissue samples.* ***A.*** *Comparison of areas of Stromal/Collagen-rich area in primary and recurrent tumors. In each ROI Stromal/Collagen-rich area normalized per total tissue area****. B.*** *Comparison of COL1A1 expression intensities in primary vs. recurrent AGCT tissue samples. Mean values per sample are plotted and used for statistical analysis. A Wilcoxon test was used to compare differences between the two AGCT conditions, with p-values displayed on the plots.*
