# Supplementary material for: Multiplexed Imaging Mass Cytometry Reveals Tumor-immune Microenvironment–dependent Hormone Receptor Expression in Adult-Type Ovarian Granulosa Cell Tumors
Source: Cancer Res Commun. 2025 Oct 27;5(10):1894–909. doi: 10.1158/2767-9764.CRC-25-0333 (PMC12555029; doi:10.1158/2767-9764.CRC-25-0333)
Supplement: Supplementary Figure S10 — Figure S10. IMC and IHC staining comparison: PR staining [file crc-25-0333_supplementary_figure_s10_suppsf10.pdf]

Supplementary Figure S10. IMC and IHC staining comparison: PR staining

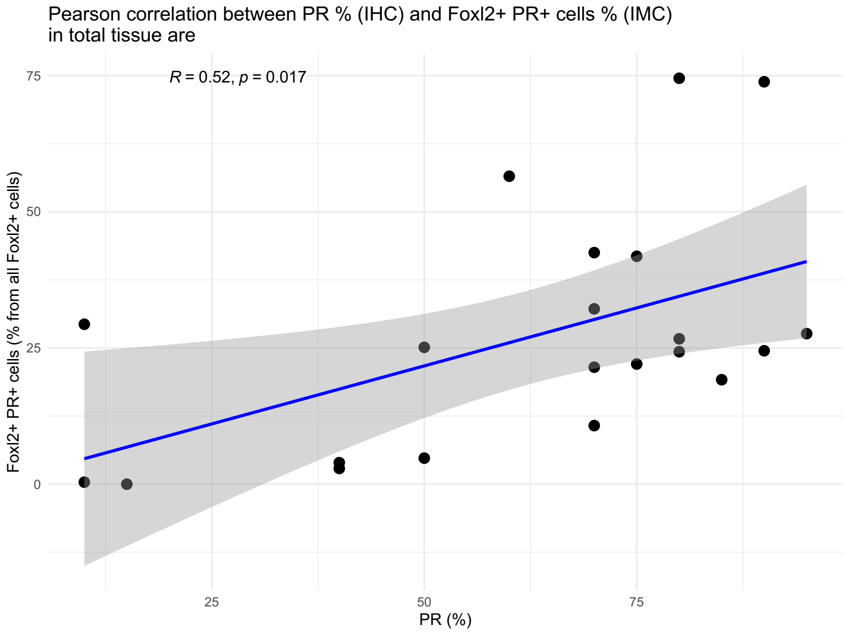

Supplementary Figure S10. *Correlation plot comparing PR (progesterone receptor) expression levels obtained from IMC analysis and IHC staining across AGCT samples, demonstrating concordance between the two methods.*
