# Supplementary material for: Multiplexed Imaging Mass Cytometry Reveals Tumor-immune Microenvironment–dependent Hormone Receptor Expression in Adult-Type Ovarian Granulosa Cell Tumors
Source: Cancer Res Commun. 2025 Oct 27;5(10):1894–909. doi: 10.1158/2767-9764.CRC-25-0333 (PMC12555029; doi:10.1158/2767-9764.CRC-25-0333)
Supplement: Supplementary Figure S11 — Figure S11. t-SNE dimensionality reduction map for Foxl2+ cells in AGCT TME [file crc-25-0333_supplementary_figure_s11_suppsf11.pdf]

**Supplementary Figure S11. t-SNE dimensionality reduction map for Foxl2+ cells in AGCT TME**

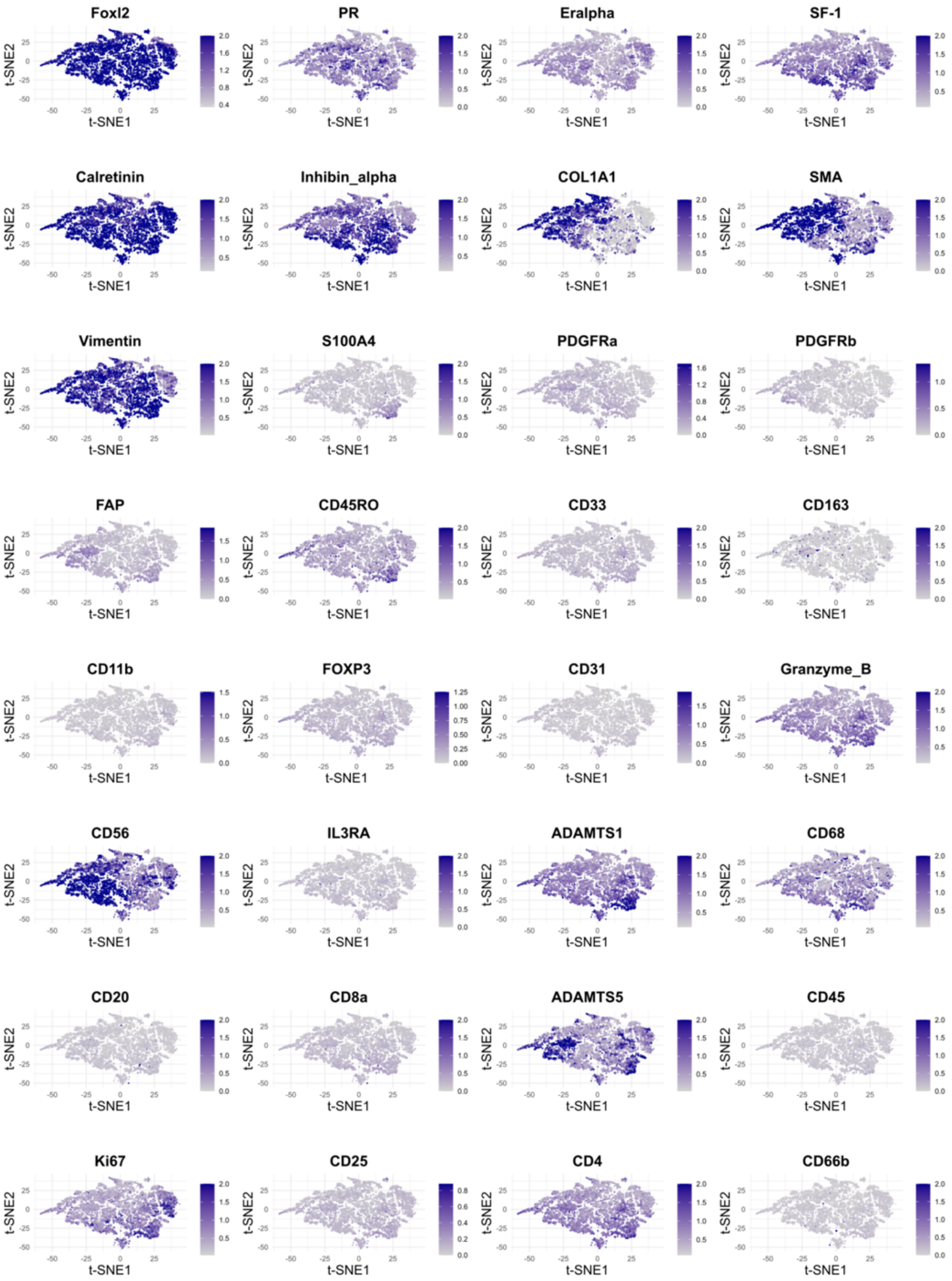


***Supplementary Figure S11.*** *t-SNE dimensionality reduction map showing the expression intensities of all markers included in the AGCT IMC panel. Each dot represents a single Foxl2$^{+}$ cell. The analysis includes eight subtypes defined by PR and ER expression in Foxl2$^{+}$ COL1A1$^{-}$ and Foxl2$^{+}$ COL1A1$^{+}$ populations: PR$^{+}$ ER$^{+}$, PR$^{+}$ ER$^{-}$, PR$^{-}$ ER$^{+}$, and PR$^{-}$ ER$^{-}$ for each group. A total of 1,000 cells were randomly selected per subtype, resulting in 8,000 cells being used for the analysis.*
