# Supplementary material for: Multiplexed Imaging Mass Cytometry Reveals Tumor-immune Microenvironment–dependent Hormone Receptor Expression in Adult-Type Ovarian Granulosa Cell Tumors
Source: Cancer Res Commun. 2025 Oct 27;5(10):1894–909. doi: 10.1158/2767-9764.CRC-25-0333 (PMC12555029; doi:10.1158/2767-9764.CRC-25-0333)
Supplement: Supplementary Figure S12 — Figure S12. Differences in fractions of Foxl2+ COL1A1-, Foxl2+ COL1A1+, and stromal cells [file crc-25-0333_supplementary_figure_s12_suppsf12.pdf]

Supplementary Figure S12. Differences in fractions of Foxl2+ COL1A1-, Foxl2+ COL1A1+, and stromal cells

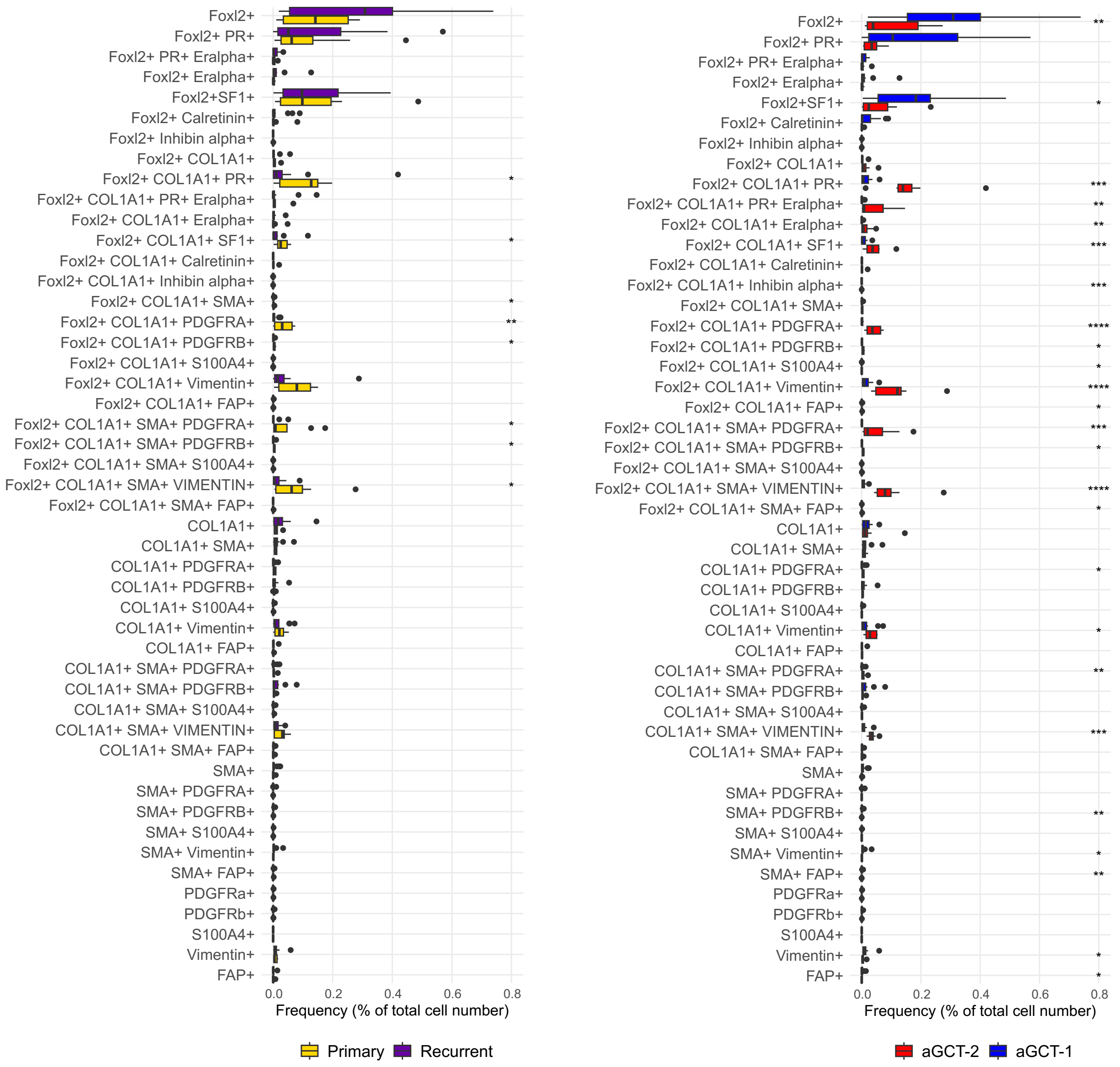

Supplementary Figure S12. *Comparison of the fractions of Foxl2⁺ cell subtypes and stromal cells between primary and recurrent AGCTs (left panel) and between AGCT-1 and AGCT-2 subtypes (right panel), highlighting greater differences in cell fractions between AGCT subtypes than between primary and recurrent samples. Cell numbers were normalized to the total number of cells per image. Each dot represents the mean cell fraction across all ROIs within a single sample. Statistical comparisons were performed using the Wilcoxon test, with p-values shown on the plots.*
