# Supplementary material for: Multiplexed Imaging Mass Cytometry Reveals Tumor-immune Microenvironment–dependent Hormone Receptor Expression in Adult-Type Ovarian Granulosa Cell Tumors
Source: Cancer Res Commun. 2025 Oct 27;5(10):1894–909. doi: 10.1158/2767-9764.CRC-25-0333 (PMC12555029; doi:10.1158/2767-9764.CRC-25-0333)
Supplement: Supplementary Figure S14 — Figure S14. Differences in fractions of immune cells in different tissue compartments of AGCT subtypes [file crc-25-0333_supplementary_figure_s14_suppsf14.pdf]

Supplementary Figure S14. Differences in fractions of immune cells in different tissue compartments of AGCT subtypes

A. Tissue area

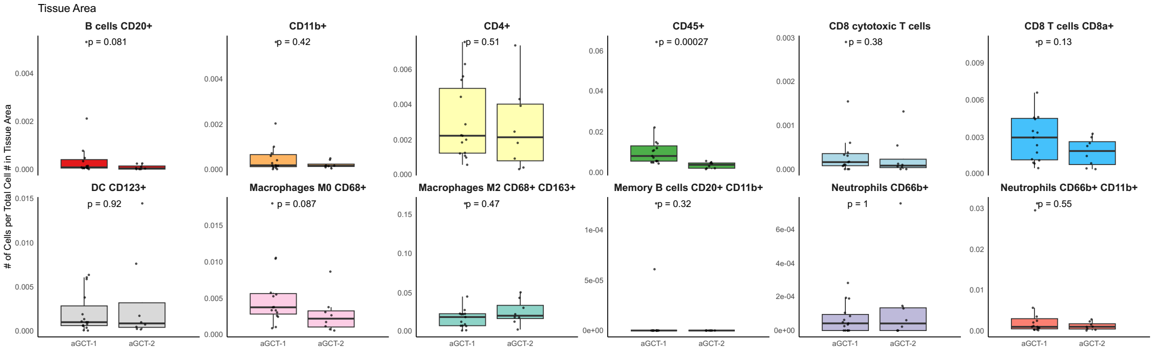

B. Tumor area

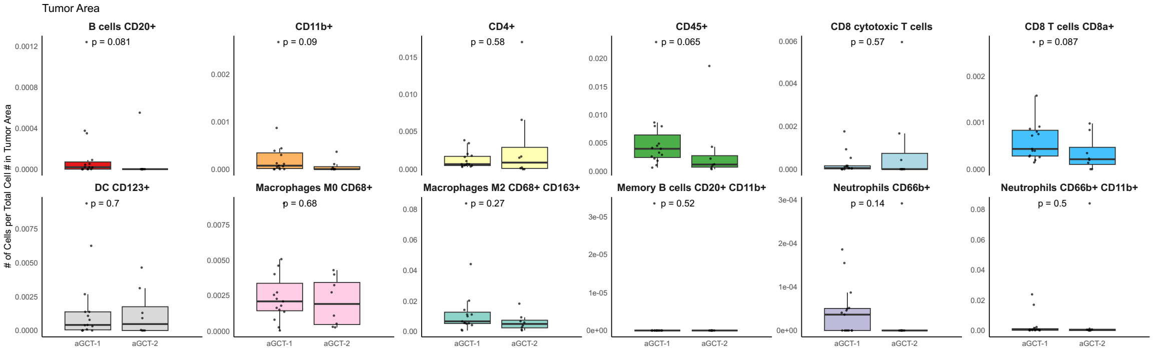

C. Stromal/Collagen-rich area

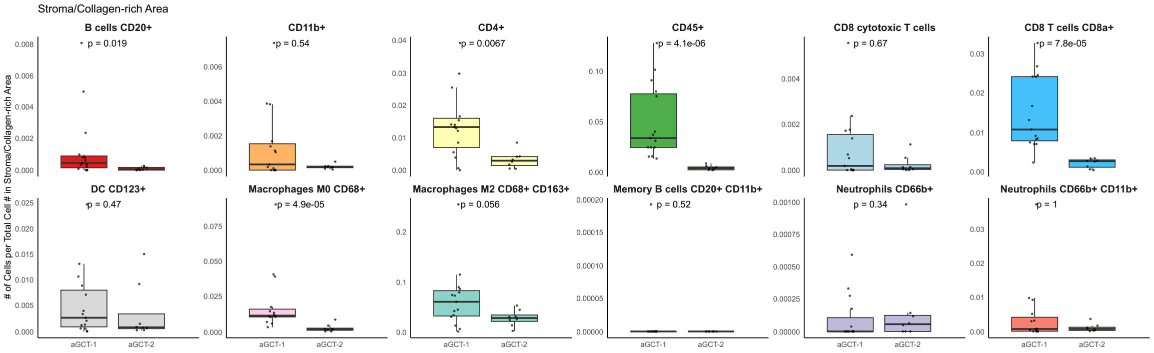

Supplementary Figure S14. *Comparison fractions of immune cell types detected AGCT-1 and AGCT-2 tumor subtypes in different compartments: total tissue, tumor area, and collagen-rich area. Cell numbers were normalized to the total number of cells per image. Each dot represents the mean cell fraction across all ROIs within a single sample. Statistical comparisons were performed using the Wilcoxon test, with p-values shown on the plots.*
