# Supplementary material for: Multiplexed Imaging Mass Cytometry Reveals Tumor-immune Microenvironment–dependent Hormone Receptor Expression in Adult-Type Ovarian Granulosa Cell Tumors
Source: Cancer Res Commun. 2025 Oct 27;5(10):1894–909. doi: 10.1158/2767-9764.CRC-25-0333 (PMC12555029; doi:10.1158/2767-9764.CRC-25-0333)
Supplement: Supplementary Figure S15 — Figure S15. Cell-cell interaction comparative analysis [file crc-25-0333_supplementary_figure_s15_suppsf15.pdf]

Supplementary Figure S15. Cell-cell interaction comparative analysis

A. AGCT-1 (top tile) vs AGCT-2 (bottom tile)

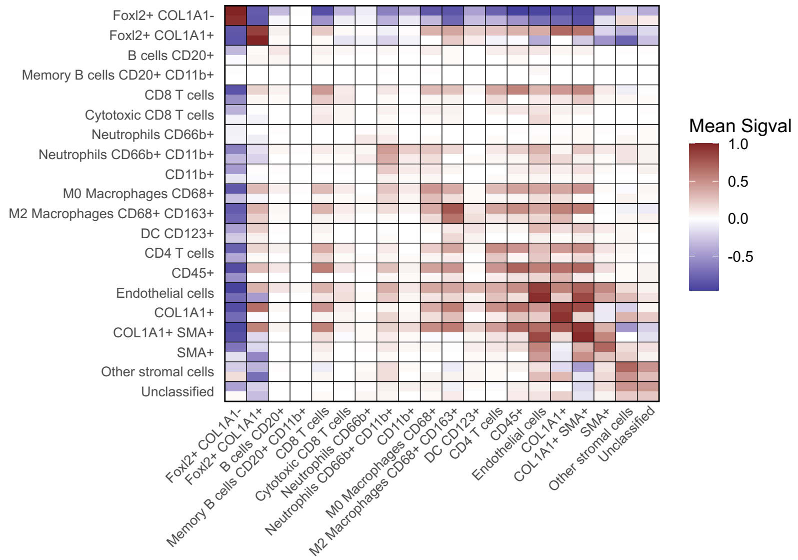

B. Primary (top tile) vs Recurrent (bottom tile) AGCTs

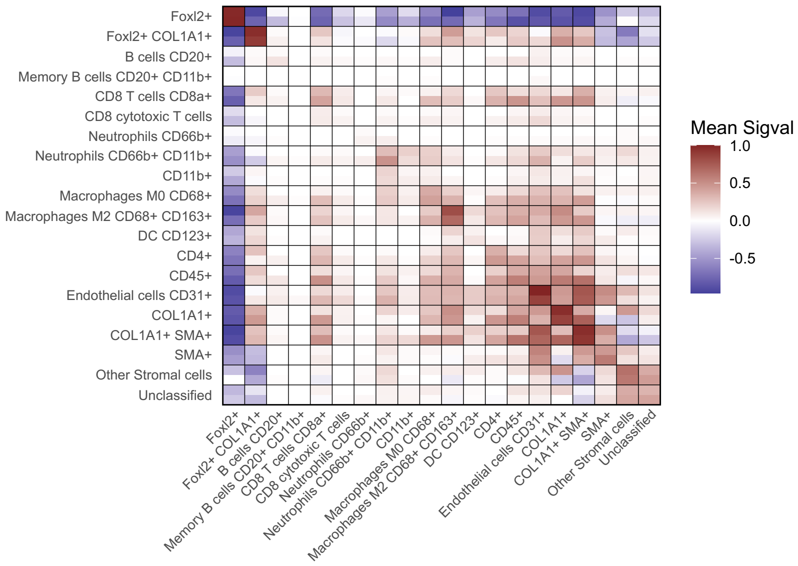

Supplementary Figure S15. *Heatmap of pairwise cellular interactions and avoidances between cell types (x-axis: from cell type; y-axis: to cell type), illustrating increased immune–stromal interactions in (A) AGCT-1 and (B) recurrent AGCTs. Interactions were calculated using the 10 nearest neighbors (knn method for interaction graph building). Statistical significance was assessed using 1,000 permutations, comparing observed interactions in each ROI to a null distribution generated by random cell-type label shuffling, with a significance threshold of 0.01. Only significant values contributed to the averaged interaction–avoidance scores across ROIs in one sample. Associations are shown in red (+1), and avoidances in blue (–1).*
