# Supplementary material for: Multiplexed Imaging Mass Cytometry Reveals Tumor-immune Microenvironment–dependent Hormone Receptor Expression in Adult-Type Ovarian Granulosa Cell Tumors
Source: Cancer Res Commun. 2025 Oct 27;5(10):1894–909. doi: 10.1158/2767-9764.CRC-25-0333 (PMC12555029; doi:10.1158/2767-9764.CRC-25-0333)
Supplement: Supplementary Figure S16 — Figure S16. Expression of IDO1, SPP1, and VEGFA genes in primary vs recurrent AGCT samples [file crc-25-0333_supplementary_figure_s16_suppsf16.pdf]

Supplementary Figure S16. Expression of *IDO1*, *SPP1*, and *VEGFA* genes in primary vs recurrent AGCT samples

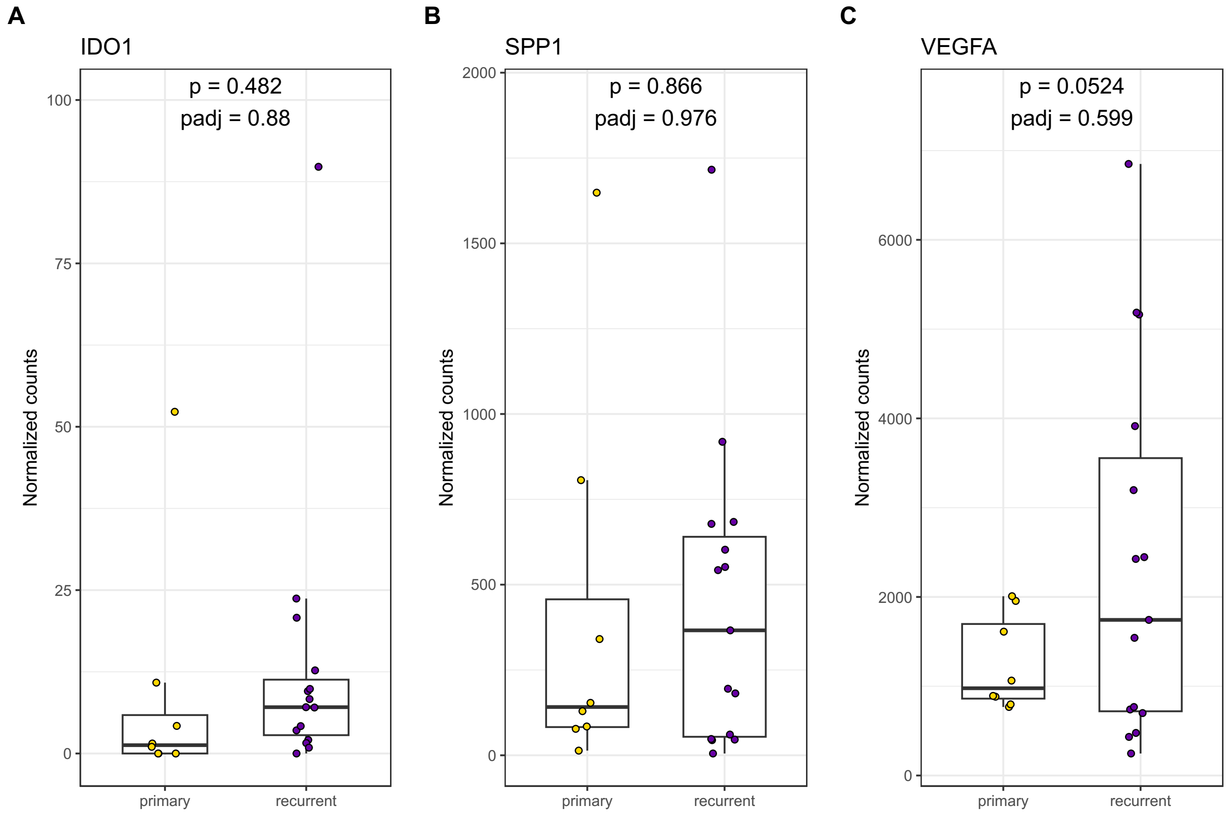

Supplementary Figure S16. *Box plots showing the expression levels of IDO1, SPP1, and VEGFA genes, which were previously identified as differentially expressed genes between primary and recurrent tumors in the Hilliard TS and Juncker-Jensen A. (2024) study. In the current study, no statistically significant differences in the expression of these genes between primary and recurrent tumors were observed. Wald test p-values (recurrent vs. primary conditions) were calculated using DESeq2 (v1.45.3), with multiple testing correction applied using the Benjamini–Hochberg method (padj). Both p-values are shown above the corresponding plots.*
